# In silico structural, phylogenetic and drug target analysis of putrescine monooxygenase from *Shewanella putrefaciens-95*

**DOI:** 10.1186/s43141-022-00338-z

**Published:** 2022-04-12

**Authors:** Anil H. Shyam Mohan, Saroja Narsing Rao, Srividya D., N. Rajeswari

**Affiliations:** 1 Department of Biotechnology, Dayananda Sagar College of Engineering, Kumaraswamy Layout, Shavige Malleswara Hills, Bengaluru-78, Karnataka India; 2 Pesticide Residue and Food Quality Analysis Laboratory, University of Agricultural Sciences, Raichur, Karnataka 584104 India; 3 Department of Biotechnology, Davangere University, Shivagangothri, Davangere, Karnataka 577007 India

**Keywords:** N-Hydroxylating monooxygenases, *Shewanella putrefaciens*, Physicochemical characteristics, Phylogenetic relationship, Molecular docking, Naturally occurring inhibitors

## Abstract

**Background:**

The enormous and irresponsible use of antibiotics has led to the emergence of resistant strains of bacteria globally. A new approach to combat this crisis has been nutritional immunity limiting the availability of nutrients to pathogens. Targeting the siderophore biosynthetic pathway that helps in iron acquisition, an essential microelement in the bacterial system has been the topic of interest in recent days that backs the concept of nutritional immunity. Supporting this view, we have chosen to study a key enzyme in the biosynthetic pathway of putrebactin called putrescine monooxygenase (*Sp*PMO) from *Shewanella putrefaciens.* In our previous study, we co-expressed putrescine monooxygenase recombinantly in *Escherichia coli* BL21 Star (DE3). The bioinformatic analysis and screening of inhibitors will broaden the scope of *Sp*PMO as a drug target.

**Results:**

In the present study, we have analysed the physicochemical properties of the target enzyme and other N-hydroxylating monooxygenases (NMOs) using ExPASy server. The target enzyme *Sp*PMO and most of the selected NMOs have a slightly acidic isoelectric point and are medially thermostable and generally insoluble. The multiple sequence alignment identified the GXGXX(N/A), DXXXFATGYXXXXP motives and conserved amino acids involved in FAD binding, NADP binding, secondary structure formation and substrate binding. The phylogenetic analysis indicated the distribution of the monooxygenases into different clades according to their substrate specificity. Further, a 3D model of *Sp*PMO was predicted using I-TASSER online tool with DfoA from *Erwinia amylovora* as a template. The model was validated using the SAVES server and deposited to the Protein Model Database with the accession number PM0082222. The molecular docking analysis with different substrates revealed the presence of a putrescine binding pocket made of conserved amino acids and another binding pocket present on the surface of the protein wherein all other ligands interact with high binding affinity. The molecular docking of naturally occurring inhibitor molecules with *Sp*PMO 3D model identified curcumin and niazirin with 1.83 and 2.81 μM inhibition constants as two promising inhibitors. Further studies on kinetic parameters of curcumin and niazirin inhibitors in vitro determined the Ki to be 2.6±0.0036 μM and 18.38±0.008 μM respectively.

**Conclusion:**

This analysis will help us understand the structural, phylogenetic and drug target aspects of putrescine monooxygenase from *Shewanella putrefaciens-*95 in detail. It sheds light on the precautionary measures that can be developed to inhibit the enzyme and thereby the secondary infections caused by them.

**Graphical abstract:**

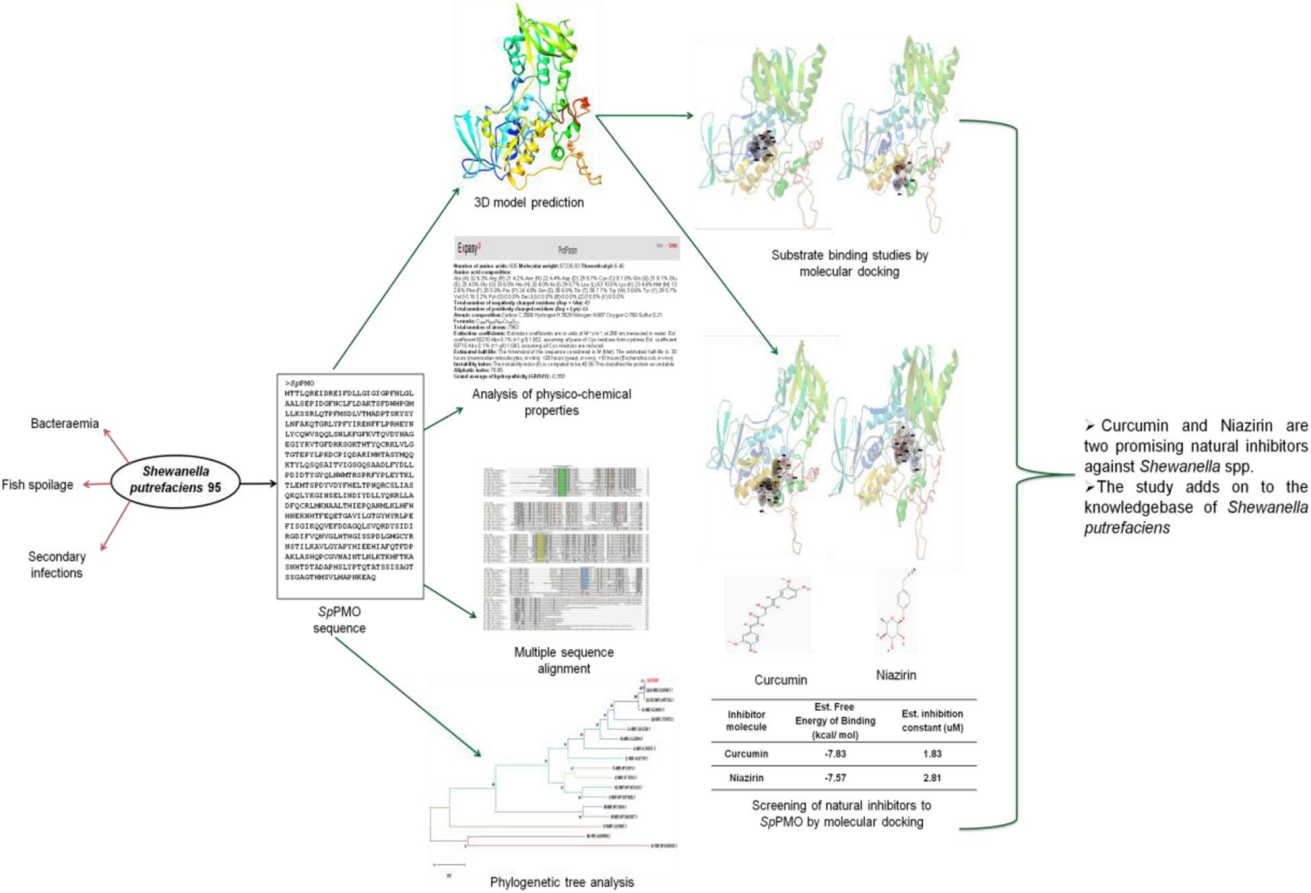

**Supplementary Information:**

The online version contains supplementary material available at 10.1186/s43141-022-00338-z.

## Highlights

- The physicochemical properties of putrescine monooxygenase from *Shewanella putrefaciens-*95 were compared with other known N-hydroxylating monooxygenases.
- The phylogenetic analysis of *Sp*PMO and other selected class B monooxygenases clearly classified NMOs according to their substrate affinity.
- The molecular docking analysis of various substrates to *Sp*PMO enzyme showed the presence of two binding pockets.
- Curcumin and niazirin were identified as two potential natural inhibitors to *Sp*PMO enzyme with 2.6±0.0036 μM and 18.38±0.008 μM inhibition constants respectively.

**Supplementary Information:**

The online version contains supplementary material available at 10.1186/s43141-022-00338-z.

## Background

Bioavailability of iron is a major challenge faced by saprophytic microorganisms. In order to tackle this, they produce iron-chelating molecules called siderophores. N-Hydroxylating monooxygenases (NMOs) are one of the principal enzymes involved in the production of hydroxamate siderophores. NMOs are classified under the category of flavin-dependent monooxygenases (FMO) and belong to class B type of FMOs [1]. NMOs are majorly present in siderophore producing prokaryotes and lower eukaryotes. Previous literature suggest that the inhibition of N-hydroxylating monooxygenases can reduce the virulence of the saprophytic pathogens [2, 3]. Therefore, NMOs can be used as drug targets.

Many studies are focussed on the heterologous expression and functional and structural characterization of NMOs from *Escherichia coli*, *Pseudomonas aeroginosa*, *Mycobacterium* spp, *Aspergillus* spp. etc. [4, 5]. The researchers have also identified methimazole, celastrol as the inhibitors to the SidA, the NMO from *Aspergillus* spp. [6, 7]. In our previous study, we have also co-expressed NMO from *Shewanella putrefaciens* (putresine monooxygenase—*Sp*PMO) along with GroES/EL chaperone in *E. coli* BL21 star (DE3) [8]. *Shewanella putrefaciens* is gram-negative bacteria usually present in shallow water bodies. It is a major cause of fish spoilage, poultry and high pH meat destruction [9]. *S. putrefaciens* along with other *Shewanella* species can be responsible for secondary infections and bacteraemia in humans [10]. Therefore, prohibition of this bacterium holds potential applications in both food industry and health sector.

Since putrescine monooxygenase plays a key role in the process of siderophore production and thereby iron uptake, the inhibition of *Sp*PMO will be a good approach for the control of *Shewanella putrefaciens*. As the heterologous expression of the NMOs in the soluble form is a major hindrance for the study of these enzymes [11], the drug target study using molecular docking tools can provide us with valuable insights. In this investigation, we intend to explore and compare the physicochemical properties, phylogenetic relationships and secondary structure analysis of *Sp*PMO with other known NMOs. We also analysed the substrate specificity of selected diamines to *Sp*PMO in silico*.* Further, we also screened a set of naturally occurring inhibitor molecules by molecular docking approach and studied the kinetic parameters of the two best inhibitors.

## Methods

### Sequence retrieval

The amino acid sequence of putrescine monooxygenase from *Shewanella putrefaciens-95* was retrieved from the previous work [8]. The other NMO sequences from *Shewanella baltica*, *Shewanella putrefaciens* 200, *Shewanella putrefaciens* CN32, *Gordonia ruberipertincta*, *Streptomyces*, *Bordetella pertussis*, *Erwinia amylovora*, *Escherichia coli*, *Mycobacterium tuberculosis*, *Nocardia facinica*, *Pseudomonas aeruginosa*, *Kutzneria* spp., *Aspergillus fumigates*, *Amycolatopsis alba* along with the representative sequences of Baeyer-Villiger monooxygenases (BVMO) from *Xanthobacter flavus* and flavin containing monooxygenases (FMO) from *Homo sapiens* and *Methylophaga aminisulfidivorans* were retrieved from the National Centre for Biotechnology Information (NCBI) website [11] (Supplementary data).

### Primary sequence analysis

The amino acid sequence of *Sp*PMO and other NMOs were analysed by Expasy ProtParam tool [12]. The amino acid composition, the isoelectric point (pI), the molecular weight (MW), extinction coefficient (EC—quantitative study of protein-protein and protein-ligand interactions), instability index, aliphatic index (AI), and grand average of hydropathicities (GRAVY) were noted. Protein solubility index is calculated by Protein-sol [13].

### Multiple sequence alignment and construction of phylogenetic tree

To construct the phylogenetic tree, the retrieved amino acid sequences of various NMOs were aligned using ClustalW [14] with default parameters. The aligned file was visualized and the conserved sequences were highlighted using GeneDoc software (version 2.7.000) [15]. The same file used as input for MEGA-X (version 10.1.7) [16] software and the evolutionary tree was constructed applying the maximum likelihood method with a confidence level of 1000 bootstrap replicates.

### 3D model prediction for *Sp*PMO

Three-dimensional structures of *Sp*PMO were constructed using Iterative Threading ASSEmbly Refinement (I-TASSER) server [17–19]. The predicted structure was verified using SAVES server [20]. The model in the specified format (.pdb) was submitted to Protein Model Database [21].

### Substrate affinity analysis using molecular docking

The chemical structures (3D structures in .sdf format) of the selected substrates were obtained from the PubChem compound database and converted to. pdbqt format. *Sp*PMO 3D model was viewed in the Autodock 4.2.6 software and the Gasteiger charges were added to the entire protein. The grid parameter file was prepared by setting the grid box to 60 Å × 60 Å × 60 Å with a spacing of 0.408 Å. The XYZ co-ordinates on the central grid point showed 64.460, 55.020 and 62.510 points. The Lamarckian genetic algorithm was employed for docking; 50 GA runs were performed for individual substrates with a population size of 300. Maximum number of evaluations was set to 2,500,000 and maximum number of generations to 27,000. All other parameters were set to default and docking was carried out using Autodock tools version 1.5.6 [22].

### Molecular docking with selected naturally occurring antimicrobial compounds

The naturally occurring inhibitors mainly consisting of aliphatic and aromatic amines (about 30) with their previously known antimicrobial properties were selected from MolPort database [23]. The chemical structures of each inhibitor (.sdf format) were downloaded from PubChem compound database. Each of the inhibitors was computed with Gasteiger charges and saved in .pdbqt format. *Sp*PMO protein was pre-processed with the addition of Gasteiger charges and saved as .pdbqt file. Later each of the inhibitor molecules were docked against *Sp*PMO protein using the same grid and docking parameters as that of substrate docking protocol.

### In vitro inhibitor studies

The quantity of N-hydroxyl putrescine formation in the presence and absence of inhibitors was assayed by a modified Csaky iodine oxidation protocol as done in our previous study [8]. The enzyme *Sp*PMO (1 μM) in 100 mM sodium phosphate buffer was incubated with putrescine (0–25 mM) and the inhibitors curcumin or niazirin (0–0.01 mM) for 10 min at 25°C and 1000 rpm shaking. The cofactor NADPH concentration was maintained constant at 2.5 mM. The reaction was aborted by the addition of 0.05 mL of 2N perchloric acid and 50 μL of this mixture was moved to a neatly labelled 96 well plate. The mixture was neutralized by the addition of 10% sodium acetate solution. The individual wells were filled with 50 μL 1% sulfanilic acid in 25% acetic acid and 20 μL of 0.5% iodide in glacial acetic acid. The plate was incubated for 10 min with shaking at 25°C. The addition of 0.1 N sodium thiosulfate eliminated excess iodine, and the colour developed on the addition of 20 μL of 0.6% α-naphthalamine after an incubation period of 45–50 min. The absorbance at 565 nm was measured using a Biorad microplate reader. The amount of product formation was calculated with the help of hydroxylamine hydrochloride standard curve. The kinetics data were fit to Michaelis Menten curve and subsequently Lineweaver Burk plots were used to obtain *K*_m_, *V*_max_, and *K*_i_.

## Results

The initial critical step of siderophore biosynthesis in microorganisms is catalyzed by N-hydroxylating monooxygenases (NMO) belonging to class B FAD-containing monooxygenases [24]. These NMO need either NAD or NADPH for catalysis [5]. In our previous study, chaperone-mediated expression of *Sp*PMO was carried out along with kinetic studies using putrescine and lysine substrates [8]. Even so, *Sp*PMO is not explored exhaustively. In the present study, physicochemical properties, evolutionary relationships, functional and structural properties and evaluation of substrate binding affinity of NMO from *Shewanella putrefaciens* (*Sp*PMO) (MH899123) have been addressed in detail using various bioinformatics tools. Further, we have also attempted to screen a few naturally occurring inhibitors against *Sp*PMO enzyme.

### Sequence retrieval and primary sequence analysis

The amino acid sequence of *Sp*PMO protein, along with seventeen known sequences of class B monooxygenases, was compared and analyzed for their physicochemical properties. This seventeen class B monooxygenases included thirteen NMOs from bacteria, fungi and actinomycetes. Two sequences represented FMOs from *Homo sapiens* and *Methylophaga aminisulfidivorans* bacteria and one representative sequence of BVMO from *Xanthomonas flavus* bacteria. The NMO sequence was chosen such that each protein had different substrate specificity and other class B monooxygenases were aptly chosen to get the right outgroups during phylogenetic analysis (Fig. 2). The results of physicochemical properties are consolidated in Table 1.

**Table 1:** Physicochemical properties of *SpPMO* and other class B monooxygenases used in the study

Accession no.	Name of the protein	Organism	No of amino acids	MW (kDa)	pI	Instability index	Solubility index	Aliphatic index	GRAVY
***Putrescine monooxygenase***									
QBX90611.1	SpPMO	*Shewanella putrefaciens 95*	505	57.24	6.46	40.56	0.143	78.85	−0.359
ACK46161.1	SbNMO	*Shewanella baltica*	501	56.76	6.24	43.51	0.154	79.48	−0.346
ADV54887.1	Sp200NMO	*Shewanella putrefaciens 200*	505	57.21	6.37	40.16	0.147	78.48	−0.359
ABP76132.1	SpCN32NMO	*Shewanella putrefaciens CN32*	505	57.22	6.31	41.97	0.147	78.48	−0.357
AOR50757.1	GrNMO	*Gordonia ruberipertincta*	438	48.84	5.03	38.24	0.394	89.66	−0.17
AGJ55094.1	StrNMO	*Streptomyces*	428	48.26	5.37	37.81	0.245	79.39	−0.356
CFO04355.1	BpNMO	*Bordetella pertussis*	478	54.95	7.32	47.78	0.081	80.86	−0.489
***Cadeverine monooxygenase***									
CBA23306.1	EaNMO	*Erwinia amylovora*	430	50.17	5.72	45.78	0.180	78.7	−0.500
***Lysine monooxygenases***									
WP_103556989.1	EcNMO	*Escherichia coli*	595	67.77	5.63	36.32	0.054	97.09	−0.094
NP_216894.1	MtNMO	*Mycobacterium tuberculosis*	431	46.94	6.17	26.65	0.227	88.54	−0.164
WP_099421877.1	NfNMO	*Nocardia farcinica*	456	49.71	7.94	33.01	0.273	89.23	−0.246
***Ornithine monooxygenases***									
NP_251076.1	PaNMO	*Pseudomonas aeruginosa*	443	49.48	6.03	39.44	0.134	90.32	−0.252
WP_043726233.1	KtzNMO	*Kutzneria* spp.	424	47.29	5.51	40.11	0.412	78.63	−0.325
XP_755103.1	AfNMO	*Aspergillus fumigates*	501	56.87	8.78	49.32	0.244	80.78	−0.523
WP_039794392.1	AlNMO	*Amycolatopsis alba*	447	49.44	5.08	34.11	0.468	86.58	−0.189
***Flavin-containing monooxygenases***									
NP_001269621.1	HsFMO	*Homo sapiens*	536	60.81	6.58	36.29	0.289	87.09	−0.053
AAM18566.2	MaFMO	*Methylophaga aminisulfidivorans*	456	52.99	5.13	33.34	0.469	65.26	−0.543
***Baeyer-Villiger monooxygenase***									
CAD10801.1	XfBVMO	*Xanthobacter flavus*	446	60.32	5.93	25.67	0.283	77.4	−0.235

The number of amino acids in all the chosen class B monooxygenases ranged from 400 to 600, indicating that many substitutions, additions and deletions would have taken place in the due course of evolution. Ornithine monooxygenase of *Aspergillus fumigatus* is the longest NMO in the group with 595 amino acids and a molecular weight of 67.77 kDa. NMO from *Shewanella* species including *Sp*PMO are generally 505 amino acids in length with a molecular weight ranging from 56.76 to 57.24 kDa. The isoelectric points (pIs) of the chosen NMOs fall in the range of 5.0–8.8; most of them are having slightly acidic pIs from 5.5 to 6.5. NMOs from *Shewanella* species also fall in the slightly acidic range (6.2–6.4). These data suggest that the proteins may be present in moderately halophytic environments [25, 26]. This also correlates well with the fact that *Shewanella* genus includes marine bacteria, most of which are found in extreme aquatic habitats [27].

Instability index (II) model was developed to calculate the in vivo protein stability based on the dipeptide composition of the primary sequence. Even though the application of this method for the calculation of protein stability under in vivo conditions is questionable using this method, it is still used routinely for prediction [28]. According to this method, proteins with instability index values greater than 40 are considered unstable. Among the protein groups chosen in our study, the instability indices of all the NMOs are close to 40 and above. In particular, the instability index of putrescine monooxygenase group has been observed to be between 37 and 48. The unstable nature of these proteins can be noted during heterologous expression studies of these proteins [4, 8, 29], wherein they have to be tagged with a soluble protein like MBP or co-expressed with a chaperone for soluble expression. Although the instability index of lysine monooxygenases falls within 40, the researchers have faced difficulties in soluble expression of these enzymes in *E. coli* [5].

The aliphatic index (AI) is directly proportional to the number and relative volume (percentage) of the aliphatic amino acids such as alanine, valine, isoleucine and leucine in the given protein. Higher value of aliphatic index indicates that the protein is highly thermostable. The AI of the chosen class B monooxygenases is in the range of 65.26 to 97.09. The FMO from *Methylophaga aminisulfidivorans* possess the lowest AI of 65.26 and the NMO from *E. coli* possess the highest AI of 97.09 indicating high thermostability among the shown NMOs. AI of *Shewanella* species is around 78 and falls in the medium range. Higher percentage of leucine, alanine, valine and isoleucine observed in the NMOs (Table S1) upholds the fact that NMOs possess high aliphatic index and are thermostable in nature. The grand average of hydropathy (GRAVY) values of all the class B monooxygenases is negative. This suggests that class B monooxygenases are polar and hydrophilic in nature [30]. The presence of both hydrophobic and hydrophilic amino acids implies that the proteins are amphipathic and may function as transmembrane proteins.

### Multiple sequence alignment and construction of the phylogenetic tree

**Fig. 1:**
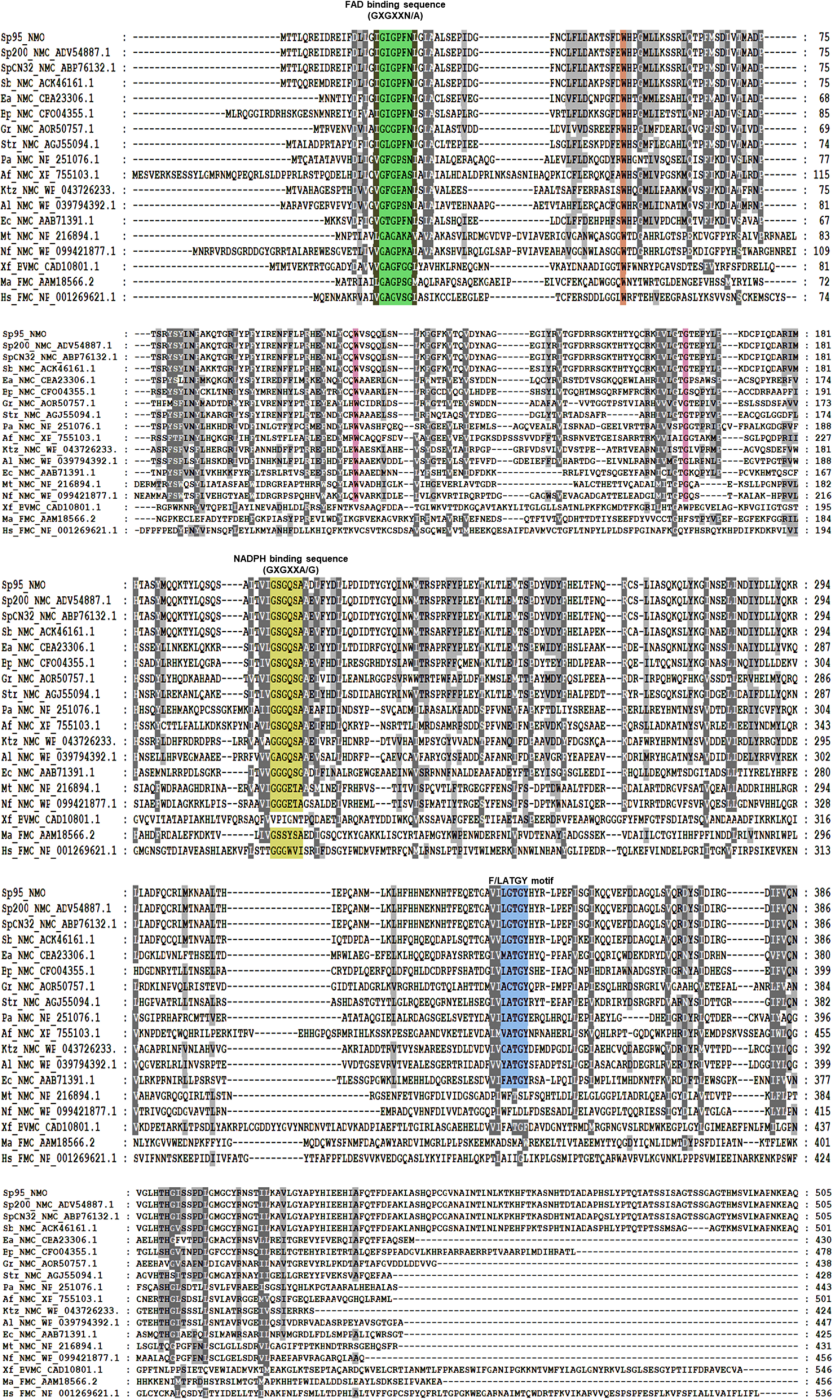
Multiple sequence alignment of the selected NMOs, FMOs and BVMO. The selected sequences (Table 1) were aligned using CLUSTAL W and the conserved sequences are highlighted using Gene Doc (Version 2.7)

The multiple amino acid sequence (Fig S1) alignment clearly shows the presence of important conserved motifs in *Sp*95_NMO and other selected N-hydroxylating monooxygenases (Fig. 1). Instead of glycine in the sixth position of FAD binding motif [31], aspargine/alanine GXGXX(N/A) is present in N-hydroxylating monooxygenases. In the NADP binding sequence also glycine at the sixth position (GXGXX**G**) is replaced by alanine (GXGXX**A**) in the majority of the NMOs (Fig. 1). Further, the characteristic FATGY motif with the sequence DXXXFATGYXXXXP is present in all the selected NMOs (Table 1), though aspartic acid (D1) and proline (P14) are not conserved. D1 is replaced by either glutamic acid (E1) or glycine (G1). In the highly conserved FATGY sequence also, phenylalanine (F) is replaced by leucine (L) and alanine is substituted by glycine in *Shewanella* and *Bordotella* species*.* Only ‘TGY’ sequence is highly conserved across the selected NMO sequence with an exception of lysine monooxygenases from *Mycobacterium tuberculosis* and *Nocardia farcinica* (Fig. 1). Along with the coloured conserved motifs, other conserved sequences visualized in the GeneDoc software are marked in grey. Tryptophan at the positions 50 and 113 in *Sp*PMO are found to be conserved across the NMOs and tryptophan (W50) is predicted to be involved in FAD binding using the COFACTOR server (Fig S8). Other amino acids like Glycine 162, Glycine 164, Leucine 399 and Valine 127 are conserved among NMOs and are involved in FAD binding. Arginine 104 and Proline 170 are involved in NADP binding (Fig S9) and conserved across all the selected NMO sequences except for lysine monooxygenases from *Mycobacterium* and *Nocardia* species. These two enzymes accept NADH as their co-factor but not NADPH and this might be the reason for not having the conserved NADP binding amino acids in their sequence [5]. Other conserved sequences like Proline 75, Proline 250, Glycine 53 and Glycine 393 are involved in secondary structure formation; nonpolar amino acids like alanine 28, Phenylalanine 65, Proline 75, Alanine 209, Leucine 215, Glycine 393 and Proline 250 are highly conserved; and these amino acids may help in the formation of pockets for ligand binding.

**Fig. 2:**
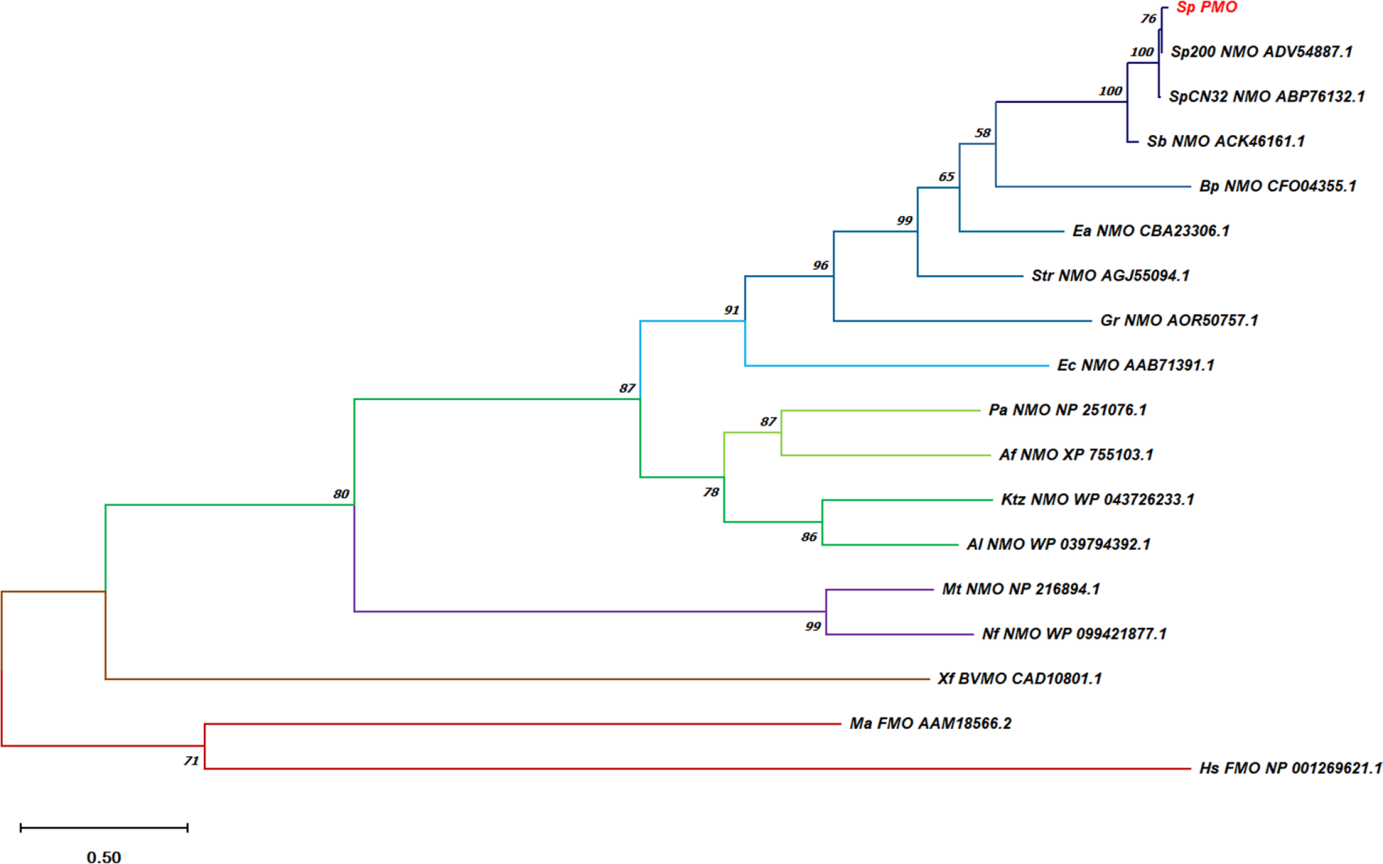
Evolutionary analysis of *SpPMO* and other class B monooxygenases by maximum likelihood method performed using MEGA-X software (Version 10.1.7)

The phylogenetic tree is reconstructed using the maximum likelihood method and shows the evolutionary relationships among class B monooxygenases supported by high bootstrap values (Fig. 2). The FMOs and the BVMO naturally stands as outgroup and all the selected NMOs are localized together in a clade with a frequency of 80%. Within the NMOs, the lysine monooxygenases from *Mycobacterium tuberculosis* and *Nocardia farcinica* forms a clade with 99% bootstrap frequency and all other NMOs from a clade with 87% bootstrap frequency (Fig S2). The ornithine monooxygenases from *Aspergillus fumigates*, *Pseudomonas aeruginosa*, *Kutzneria species* and *Amycolatopsis alba* are placed in one clade with 78% bootstrap support. The other sequences encoding putrescine monooxygenases, cadeverine monooxygenase and lysine monooxygenase from *E. coli* form another clade with 91% bootstrap support. The putrescine monooxygenases from *Shewanella* species are grouped together with 100% bootstrap support. The sequences from *Shewanella putrefaciens* 95 and *S. putrefaciens* 200 with 99% identity are placed together with 76% bootstrap support. From the phylogenetic tree (Fig. 2), it is clear that the evolutionary changes have taken place in the amino acid sequences of the class B Flavin containing monooxygenases based on their substrate specificity. Therefore, the amino acid substitutions are more likely to be present in the substrate binding sequences of the NMOs.

### Analysis of substrate affinity using Autodock 4.2.6

**Fig. 3:**
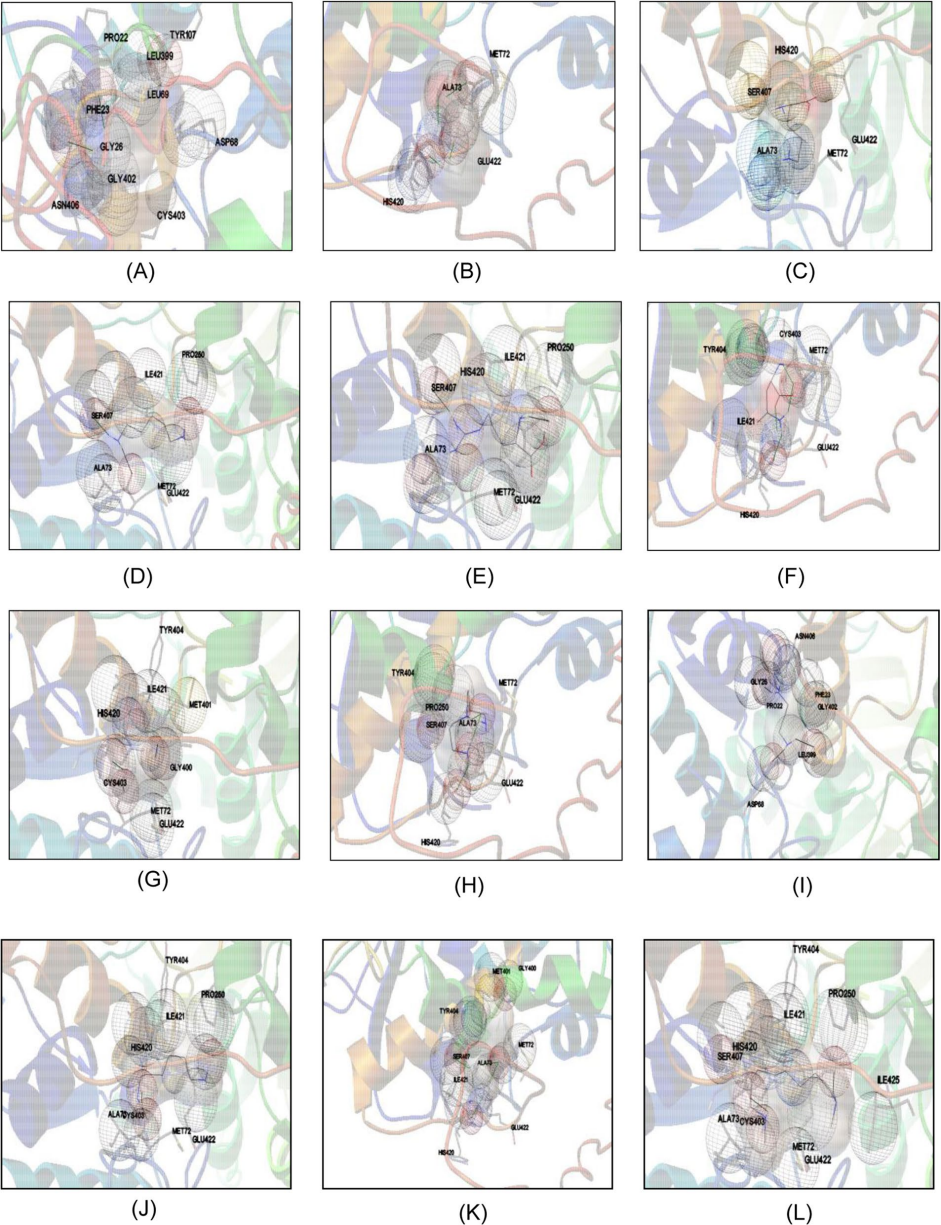
Pictorial representation of the *Sp*PMO and substrate interactions as predicted using Autodock 4.2. **A** Putrescine. **B** Lysine. **C** Ornithine. **D** Cadaverine. **E** Arginine. **F** Aspargine. **G** Glutamine. **H** Spermidine. **I** 1,6-Hexanediamine. **J** Heptanediamine. **K** Dimethyl octanediamine. **L** 1,3-Diaminopropane

Firstly, the secondary structure of *Sp*PMO was predicted using Chou-Fasman algorithm (Fig S3) and subsequently predicted the 3D-structure of *Sp*PMO that was validated using ZLab server (Fig S4), ProSA (Fig S5) and SAVES server (Fig S10) which was deposited to Protein Model Database (PMDB) after checking the overall quality factor of *Sp*PMO protein assessed using ERRAT software in SAVES sever and was found to be 84.306 (Fig S6), with a unique Accession no. PM0082222. NMOs catalyze the N-hydroxylation of the nucleophilic terminal amine groups of the primary aliphatic diamines. The best poses of interaction between selected aliphatic diamine substrates and *Sp*PMO predicted using Autodock 4.2.6 are depicted in Fig. 3.

The docking of substrate molecules with *Sp*PMO protein showed the presence of two probable binding sites. Putrescine (1,4-Diaminobutane) and 1,3-Diaminopropane share common binding sites involving interactions with Pro22, Phe23, Gly26, Leu399, Gly402 and Asn406 (Fig. 3A, I). In the case of other substrates, Met72, Ala73, Glu422, His420, Ser407 and Tyr407 are commonly involved (Fig. 3B–H, J–L). Though cadaverine is also an aliphatic diamine sharing similar chemical structure to that of putrescine with one additional carbon atom, the binding sites are not the same. The visual observation of the docked poses of the selected substrates suggests that a four-carbon chain aliphatic compound is the maximum length of the substrate that can fit into the substrate binding pocket. The other selected substrates interact with the outer surface amino acids with Vander Waals forces, electrostatic interactions and hydrogen bonding but cannot move into the binding pocket situated deep inside, so that it comes to the vicinity of FAD and NADP binding regions and participate in the oxidative and reductive cycles of N-hydroxylation activity of the *Sp*PMO enzyme. The free energy of binding and dissociation constant as estimated by the software is lower for arginine with −4.79 kcal/mol and 309.71 μM respectively. It is followed by Dimethyloctanediamine > heptanediamine > ornithine > spermidine > glutamine > lysine > 1, 6-hexanediamine > putrescine > cadaverine > 1,3-diaminopropane (Table 2). Despite the high binding affinity of the other substrates, they might be involved only in the oxygenase activity of the enzyme and do not get N-hydroxylated, as they cannot fit in the binding pocket.

**Table 2:** Free energy of binding and dissociation constants of the selected substrates with *Sp*PMO

Substrates	Estimated free energy of binding (kcal/ mol)	Estimated dissociation constant	Amino acids involved in interaction with the ligand
Putrescine	−3.68	2.00 mM	Pro22, Phe23, Gly26, Asp68, Leu69, Tyr107, Leu399, Gly402, Cys403, Asn406
Lysine	−3.85	1.50 mM	Met72, Ala73, His420, Glu422
Ornithine	−4.30	701.78 μM	Met72, Ala73, Ser407, His420, Glu422
Cadaverine	−3.52	2.64 mM	Met72, Ala73, Pro250, Ser407, Ile421, Glu422
Arginine	−4.79	309.71 M	Met72, Ala73, Pro250, Ser407, Ile421, His420, Glu422
Aspargine	−3.87	1.45 mM	Met72, Cys403, Tyr404, His420, Ile421, Glu422
Glutamine	−3.98	1.21 mM	Met72, Gly400, Met401, Cys403, Tyr404, His420, Ile421, Glu422
Spermidine	−4.11	976.58 μM	Met72, Ala73, Pro250, Tyr404, Ser407, His420, Glu422
1,3-Diaminopropane	−3.02	6.08 mM	Pro22, Phe23, Gly26, Asp68, Leu399, Gly402, Asn406
1,6-Hexanediamine	−3.70	1.94 mM	Met72, Ala73, Pro250, Cys403, Tyr404, His420, Ile421, Glu422
Heptanediamine	−4.60	421.39 μM	Met72, Ala73, Gly400, Met401, Tyr404, Ser407, His420, Ile421, Glu422
Dimethyl Octanediamine	−4.69	367 μM	Met72, Ala73, Pro250, Tyr404, Cys403, Ser407, His420, Ile421, Glu422, Ile425

Predominantly the N-hydroxylation catalyzing enzymes are substrate specific while others are more relaxed [32]. Likewise, *Sp*PMO also cannot be categorized as an enzyme with broad specificity.

### Molecular docking with selected naturally occurring antimicrobial compounds

The binding of the aliphatic diamines like arginine, ornithine, spermidine, etc., with high affinity at a site other than that of putrescine binding pocket inspired us to look for the binding of other naturally occurring inhibitory molecules. A set of 30 naturally occurring inhibitor molecules mainly consisting of aliphatic and aromatic amines along with certain other bioactive phytochemicals were chosen from the MolPort natural compound database. All the molecules were individually docked against *Sp*PMO protein with the same parameters used for substrate docking.

In the selected molecules, curcumin has the highest binding affinity to the *Sp*PMO with an estimated free energy of binding of −7.83 kcal and inhibition constant of 1.83 μM. β-Alanine has the lowest affinity with the estimated free energy of binding of −3.36 kCal and 3.46 mM inhibition constant (Table 3). The visualization of the docked poses and the interaction of the inhibitors with the protein suggest that niazirin, caffeine, piperidine and betaine bind at or near the putrescine binding pocket, while the other inhibitors have a different binding site (Fig S10). Therefore, compared to curcumin, niazirin would be regarded as a best inhibitor molecule with binding free energy of −7.57 kCal and inhibition constant of 2.81 μM. Niazirin, caffeine and piperidine being aromatic amines fit in the putrescine binding pocket and therefore may behave as good competitive inhibitors of the enzyme *in vivo* condition.

**Table 3:** Free energy of binding and dissociation constants of the selected inhibitors with *Sp*PMO

Ligands	Estimated free energy of binding (kcal/ mol)	Est. inhibition constant	Amino acids involved in interaction with the ligand
Curcumin	−7.83	1.83 μM	Thr63, Pro64, Ser67, Met72, Ala73, Pro250, Gly400, Met401, Tyr404, Ser407, His420, Ile421, Glu422.
Vanilin	−4.40	591.20 μM	Met72, Ala73, Gly400, Tyr404, Ser407, His420, Ile421, Glu422
Caffeine	−5.31	128.73 μM	Gly21, Pro22, Phe23, Asn24, Gly162, Gly164, Thr165, Gln385, Asn386
Theobromine	−5.52	90.13 μM	Met72, Ala73, Pro250, Tyr404, Ser407, His420, Ile421, Glu422.
Theophylline	−5.31	127.43μM	Met72, Pro250, Met401, Cys403, Tyr404, Glu422, Ile425.
Allicin	−4.64	394.32 μM	Met72, Ala73, Pro250, Tyr404, Ser407, Tyr419, His420, Ile421, Glu422
Niazirin	−7.57	2.81 μM	Leu61, Gln62, Thr63, Leu238, Leu245, Tyr275, Pro397, Asp398, Leu399
Niazirinin	−6.08	34.77μM	Thr63, Met72, Gly400, Met401, Tyr404, Glu422
Capsaicin	−6.32	23.29 μM	Met72, Ala73, Asp74, Pro75, Thr76, Pro250, Tyr404, His420, Ile421, Glu422
Piperine	−7.75	2.07 μM	Pro64, Met72, Leu245, Thr248, Asp398, Gly400, Met401, Tyr404, Ile421, Glu422
Betaine	−3.78	1.69 mM	Pro22, Phe23, Gly26, Leu69, Leu399. Gly402, Cys403, Asn406
Dopamine	−4.58	442.35μM	Met72, Ala73, Gly400, Met401, Cys403, Tyr404, Ser407, Ile42, Glu422
Piperidine	−4.38	612.92 μM	Pro22, Phe23, Asp68, Leu69, Leu399, Gly402, Cys403, Asn406
4-Hydroxyisoleucine	−4.44	552.20 μM	Met72, Pro250, Tyr404, His420, Ile421, Glu422.
(10)-Gingerol	−5.75	61.34 μM	Met66, Met72, Ala73, Pro250, Gly400, Met401, Cys403, Tyr404, Ser407, Tyr419, His420, Glu422.
Tropine	−5.85	51.74 μM	Pro22, Phe23, Gly26, Asp68, Leu69, Tyr107, Leu399, Gly402, Cys403, Asn406
S-Allyl-L-Cysteine	−3.96	1.26 mM	Met72, Ala73, Cys403, Ser407, His420, Ile421, Glu422
Carnosine	−5.37	115.50 μM	Met72, Ala73, Cys403, Ser407, His420, Ile421, Glu422
Leonurine	−5.43	105.22μM	Met66, Met72, Ala73, Leu245, Thr248, Pro250, Asp398, Gly400, Met401, Tyr404, His420, Glu422.
Cytisine	−6.35	22.08 μM	Met72, Ala73, Pro250, Tyr404, Ser407, His420, Ile421, Glu422
Gramine	−5.85	54.74 μM	Met72, Ala73, Pro75, Tyr404, Ser407, His420, Ile421, Glu422
Tryptamine	−6.15	30.94 μM	Met72, Ala73, Tyr404, Ser407, His420, Ile421, Glu422
Beta-Pinene	−5.63	75.06 μM	Met72, Ala73, Tyr404, His420, Ile421, Glu422
Beta-Alanine	−3.36	3.46 mM	Met72, Ala73, Tyr404, Ser407, His420, Ile421, Glu422
L-Abrine	−6.48	17.76 μM	Met72, Ala73, Tyr404, Ser407, His420, Ile421, Glu422
Undecanoic acid	−4.52	483.91μM	Met72, Ala73, Pro250, Gly400, Met401, Tyr404, His420, Ile421, Glu422
Ethyl caffeate	−5.05	200.16μM	Met72, Ala73, Pro250, Gly400, Met401, Tyr404, Ser407, Tyr419, His420, Ile421, Glu422
Alliin	−4.81	296.22μM	Met72, Ala73, Gly400, Tyr404, Ser407, His420, Ille421, Glu422

### In vitro inhibitor studies

**Fig. 4:**
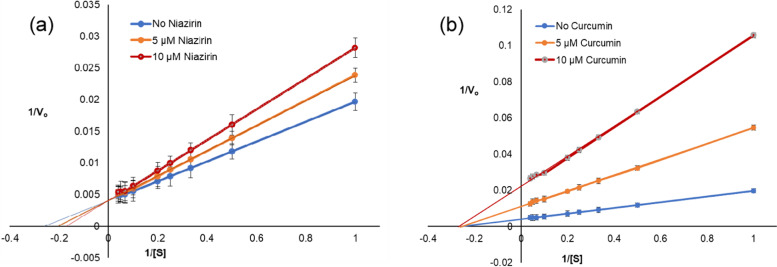
Double reciprocal plots for the inhibitors **a** niazirin and **b** curcumin

The molecular docking experiments resulted in recognizing two good inhibitors namely niazirin and curcumin. Niazirin is a bioactive compound extracted from *Moringa oleifera* while the bright yellow compound and a principal curcuminoid called curcumin is extracted from *Curcuma longa*. These naturally occurring antimicrobials were tested in vitro for their inhibitory actions against *Sp*PMO, the key enzyme for putrebactin synthesis. The initial velocities obtained with and without the inhibitors were fit to the double reciprocal plot (Fig. 4) and the kinetic constants *V*_max_ and *K*_m_ were determined (Table 4). The constant *V*_max_ and decreased *K*_m_ observed with and without niazirin suggested that it could act as a competitive inhibitor with *K*_i_ 18.38±0.008 μM. The curcumin showed decrease in *V*_max_ suggesting that it inhibits *Sp*PMO non-competitively. The *K*_i_ was determined to be 2.6±0.0036 μM. The values obtained by molecular docking correlated well with the in vitro determined values.

**Table 4:** Kinetic parameters of *Sp*PMO with and without inhibitors

	Without inhibitor	+Niazirin (5 μM)	+Niazirin (10 μM)	+Curcumin (5 μM)	+Curcumin (10 μM)
*V*_max_ (nmol min^−1^ mg^−1^)	245.59	245.59	245.55	91.49	44.86
*K*_m_ (μM)	3.81	4.84	5.89	3.98	3.71
*K*_cat_ (min^−1^)	14.24	14.24	14.24	5.306	2.602
*K*_i_ (μM)	-	18.46	18.29	2.96	2.23

Putrescine monooxygenase from *Shewanella putrefaciens* (*Sp*PMO) is a principal enzyme in the pathway of putrebactin synthesis. The role of genes coding enzymes involved in the putrebactin synthesis pathway is noted [24]. With this background, we co-expressed *Sp*PMO along with pGroES/EL chaperone in *E. coli* BL21 Star (DE3) [8]. *Sp*PMO, a putrescine hydroxylating monooxygenase, is the key enzyme of putrebactin synthesis. Thus, inhibition of *the Sp*PMO enzyme leads to the decrease of *S. putrefaciens* growth*.* Disruption of N-hydroxylating monooxygenase of the siderophore pathway leads to the decreased persistence and colonization of pathogens [2, 3]. In this study, we have chosen a set of 28 naturally occurring small molecules mainly consisting of aliphatic and aromatic amines along with certain other bioactive phytochemicals from the MolPort database to check its inhibitory activity against *Sp*PMO. We initially screened these molecules for better inhibition using docking tool Autodock version 1.5.6. In this screening, niazirin and curcumin came out as the best inhibitors based on the estimated free energy of binding and estimated inhibition constants (Table 3).

**Fig. 5:**
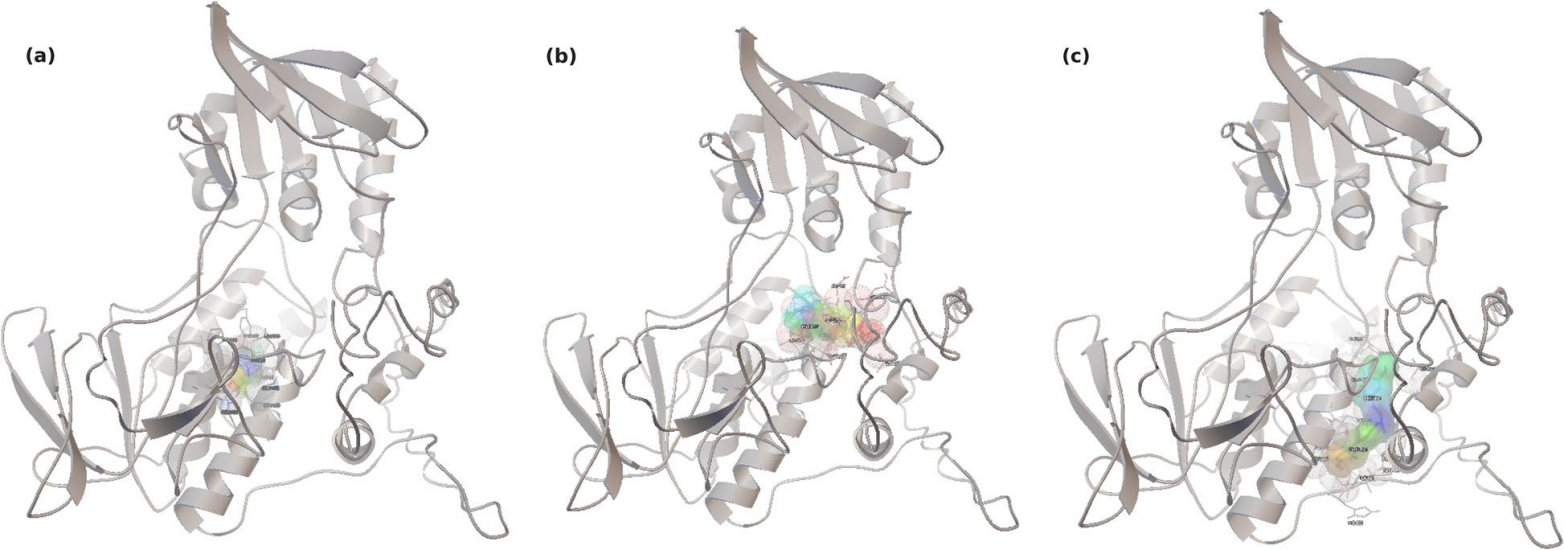
Pictorial representation of the docked poses of ligands with *Sp*PMO. **a** Putrescine. **b** Niazirin. **c** Curcumin

To further understand the inhibition by curcumin and niazirin, a kinetic study using product formation assay and double reciprocal plots was carried out. The observed *K*_m_ and *V*_max_ with and without inhibitors suggest that the curcumin inhibits *Sp*PMO non-competitively and niazirin inhibits *Sp*PMO by competitive mode of inhibition (Table 4 and Fig. 4). The data from docking studies also comply with the obtained results. The docked poses of *Sp*PMO with curcumin and niazirin (Fig S9) suggest that niazirin binds to *Sp*PMO at the vicinity of the putrescine binding pocket and therefore competes with the substrate and inhibit *Sp*PMO activity. Similarly to niazirin, curcumin does not bind to the putrescine binding pocket and curcumin competes with the substrate. It inhibits *Sp*PMO activity by non-competitive mode of inhibition (Fig. 5). In our previous study, after overexpression *Sp*PMO protein was confirmed by gene sequencing and Western blotting [8]. As curcumin and niazirin are small organic molecules, they cannot be identified by the Western blot membrane. Curcumin and niazirin inhibit *Sp*PMO protein by non-competitive and competitive modes only. In vitro, kinetic and biochemical studies show that regulatory genes are not involved in the inhibition mechanism.

## Discussion

In this study, we have explored functional and structural properties of putrescine monooxygenase from *S. putrefaciens* using distinct bioinformatic tools such as Expasy, MEGA-X, I-TASSER and Autodock. We have also explored and compared the primary structure of *Sp*PMO with other known NMOs. With the help of these tools, we were able to categorize the NMOs according to their substrate specificity (Table 1) as putrescine monooxygenases, cadaverine monooxygenases, lysine monooxygenases and ornithine monooxygenases. *Sp*PMO aptly falls within the category of putrescine monooxygenases along with *Sb*NMO, *Sp*200NMO, *Sp*CN32NMO, *Gr*NMO, *Str*NMO and *Bp*NMO. Among these NMOs, only *Sp*PMO and *Gr*NMO have been expressed recombinantly in *E. coli.* Crystallographic structure of putrescine monooxygenases has not yet been elucidated. *Sp*PMO was found to be moderately unstable, fairly thermostable and has a negative GRAVY value which suggests the possibility of protein being attached to the membrane. Our previous study of expressing *Sp*PMO recombinantly supports this data [8]. The not-so-stable nature and the difficulties faced during the recombinant expression of *Sp*PMO inspired us to develop a 3D protein model using the online ab initio modelling tool such as I-TASSER. The 3D model was derived comparing cadaverine monooxygenase from *Erwinia amylovora* as the template.

The phylogenetic tree analysis using MEGA-X software clearly demarcates all the putrescine and cadaverine monooxygenases to one clade, while ornithine and lysine monooxygenases fall into another (Fig. 2). The multiple sequence alignment using GeneDoc software (Fig. 1) identifies many conserved motifs like FAD binding, NADP binding and FATGY motifs. It also revealed the presence of other conserved amino acids like Pro22, Phe23, Asp68 and Leu399. With this information and the predicted 3D model, we further investigated the interactions of various diamine substrates with *Sp*PMO protein. The molecular docking experiment deduced two binding pockets in *Sp*PMO. One of them binds putrescine and 1,3-diamino propane and exists closer to the FAD binding region. The other pocket binds all other selected diamine substrates such as lysine, ornithine, cadaverine, spermidine and others (Table 2). Putrescine and 1,3-diamino propane interact with *Sp*PMO at the conserved amino acid regions like Pro22, Phe23, Asp68, Leu399, Gly402 and Asn406. The other substrates form bonds with Met72, Ala73, Tyr404, Ser407, His420, Ile421 and Glu422 which are at the surface of the protein. These interactions indicate that *Sp*PMO may be specific to putrescine and 1,3-diaminopropane. The other substrates such as arginine and lysine in spite of high binding energy and the lower dissociation constants (Table 2) might not get hydroxylated and increase the oxidase activity of the enzyme [5].

The putrescine monooxygenase being the first enzyme in the putrebactin biosynthetic pathway can become a potential drug target candidate. Hence, we further examined the binding interactions for a set of naturally occurring inhibitor molecules screened from MolPort natural compound database. Binding energy calculations obtained from the Autodock 4.2.6 software identified curcumin, an antimicrobial compound to bind *Sp*PMO with lowest binding energy and dissociation constant. However, the analysis of molecular interactions suggests that niazirin, a bioactive nitrile glycoside from *Moringa oleifera* [33], interacts with *Sp*PMO at the putrescine binding pocket with a binding energy of −7.57 kCal. In order to investigate further on the kinetic parameters of the two best inhibitors screened by molecular docking, we used the product formation assay and double reciprocal plots. It was evidently observed that curcumin inhibits *Sp*PMO non-competitively and niazirin inhibits *Sp*PMO competitively by the double reciprocal plots. This concept correlated well with the molecular interactions that were witnessed using Autodock. The curcumin binds to *Sp*PMO at a site other than the substrate binding and cause inhibition non-competitively, while niazirin binds at the putrescine binding pocket and inhibits the *Sp*PMO enzyme by the competitive method (Fig. S10).

## Conclusions

The present study explores the physicochemical and functional properties of putrescine monooxygenase from *S. putrefaciens* using Bioinformatic approach. The study of kinetic parameters of the inhibitor’s curcumin and niazirin has provided us with a prototype for the use of natural chemicals against severe bacterial infections. This investigation can accelerate the process of developing therapeutic and inhibitory agents against *Shewanella* species and thus avoid secondary infection and food spoilage.

## Supplementary Information

**Additional file 1: Fig. S1**. Amino acid sequences of all the proteins used for comparative study in FASTA format. **Fig. S2**. Graphical representation of percentage of helices, sheets and turns of N-hydroxylating monooxygenases from the selected microorganisms. **Fig. S3**. Pictorial representation of secondary structure predicted using Chou Fasman algorithm. **Fig. S4**. Validation of 3D model using Ramachandran plot by ZLab server. **Fig. S5**: Overall model quality assessment by ProSA-web. **Fig. S6**. Overall quality factor of *Sp*PMO95 protein assessed using ERRAT software in SAVES server and was found to be 84.306. **Fig. S7**. The overall quality of *Sp*PMO95 assessed by Verify-3D in SAVES server showing 83.96% of the residues have averaged 3D-1D score >= 0.2. **Fig. S8**. Predicted FAD binding site of *Sp*PMO, coloured in blue. FAD binding sites are given in the box. **Fig. S9**. Predicted NAD binding site of *Sp*PMO, colored in blue. NAD binding sites are given in the box. **Fig. S10**. Pictures showing the interaction of *Sp*PMO and the respective inhibitors. **Table S1**. Percentage of amino acids present in the class B monooxygenases selected for the study.

## Data Availability

All data generated or analysed during this study are included in this published article [and its supplementary information files].

## Abbreviations

*Sp*PMO: Putrescine monooxygenase from *Shewanella putrefaciens*
NMOs: N-Hydroxylating monooxygenases
FMO: Flavin-dependent monooxygenases
BVMO: Baeyer-Villiger monooxygenases
NCBI: National Centre for Biotechnology Information
pI: Isoelectric point
MW: Molecular weight
EC: Extinction coefficient
AI: Aliphatic index
GRAVY: Grand average of hydropathicities
I-TASSER: Iterative Threading ASSEmbly Refinement server
