# Supplementary material for: In silico structural, phylogenetic and drug target analysis of putrescine monooxygenase from Shewanella putrefaciens-95
Source: J Genet Eng Biotechnol. 2022 Apr 12;20:57. doi: 10.1186/s43141-022-00338-z (PMC9005580; doi:10.1186/s43141-022-00338-z)
Supplement: Supplementary file 1 — Additional file 1: Fig. S1. Amino acid sequences of all the proteins used for comparative study in FASTA format. Fig. S2. Graphical representation of percentage of helices, sheets and turns of N-hydroxylating monooxygenases from the selected microorganisms. Fig. S3. Pictorial representation of secondary structure predicted using Chou Fasman algorithm. Fig. S4. Validation of 3D model using Ramachandran plot by ZLab server. Fig. S5: Overall model quality assessment by ProSA-web. Fig. S6. Overall quality factor of SpPMO95 protein assessed using ERRAT software in SAVES server and was found to be 84.306. Fig. S7. The overall quality of SpPMO95 assessed by Verify-3D in SAVES server showing 83.96% of the residues have averaged 3D-1D score >= 0.2. Fig. S8. Predicted FAD binding site of SpPMO, coloured in blue. FAD binding sites are given in the box. Fig. S9. Predicted NAD binding site of SpPMO, colored in blue. NAD binding sites are given in the box. Fig. S10. Pictures showing the interaction of SpPMO and the respective inhibitors. Table S1. Percentage of amino acids present in the class B monooxygenases selected for the study. [file 43141_2022_338_MOESM1_ESM.doc]

***In silico* analysis of structural, phylogenetic and drug target aspects of putrescine monooxygenase from *Shewanella putrefaciens-95***

**Supplementary data**

**Fig S1. Amino acid sequences of all the proteins used for comparative study in FASTA format**

>Xf_BVMO_CAD10801.1

MTMTVEKTRTGGADYDAVVVGAGFGGLYAVHKLRNEQGMNVKAYDNAADIGGTWFWNRYPGAVSDTESFVYRFSFDRELLQRGRWKNRYVTQPEILAYLNEVADHLDLRRSYEFNTKVSAAQFDDATGLWKVTTDKGQAVTAKYLITGLGLLSATNLPKFKGMDTFKGRILHTGAWPEGVELAGKRVGIIGTGSTGVQVITATAPIAKHLTVFQRSAQFVVPIGNTPQDAETIARQKATYDDIWKQVKSSAVAFGFEESTIPAETASPEERDRVFEAAWQRGGGFYFMFGTFSDIATSQVANDAAADFIKRKLKQIVKDPETARKLTPSDLYAKRPLCGDDYYGVYNRDNVTLADVKADPIAEFTLTGIRLASGAEHELDVVIFATGFDAVDGNYTRMDMRGRNGVSLRDMWKEGPLGYLGIMEAEFPNLFMILGPNGPFTNLPPSIETQVEWIADMVKTMEAKGLKTSEPTAQARDQWVELCRTIANMTLFPKAESWIFGANIPGKKNTVMFYLAGLGNYRKVLSGLSESGYPTIIFDRAVECVA

>Ma_FMO_AAM18566.2

MATRIAILGAGPSGMAQLRAFQSAQEKGAEIPELVCFEKQADWGGQWNYTWRTGLDENGEPVHSSMYRYLWSNGPKECLEFADYTFDEHFGKPIASYPPREVLWDYIKGRVEKAGVRKYIRFNTAVRHVEFNEDSQTFTVTVQDHTTDTIYSEEFDYVVCCTGHFSTPYVPEFEGFEKFGGRILHAHDFRDALEFKDKTVLLVGSSYSAEDIGSQCYKYGAKKLISCYRTAPMGYKWPENWDERPNLVRVDTENAYFADGSSEKVDAIILCTGYIHHFPFLNDDLRLVTNNRLWPLNLYKGVVWEDNPKFFYIGMQDQWYSFNMFDAQAWYARDVIMGRLPLPSKEEMKADSMAWREKELTLVTAEEMYTYQGDYIQNLIDMTDYPSFDIPATNKTFLEWKHHKKENIMTFRDHSYRSLMTGTMAPKHHTPWIDALDDSLEAYLSDKSEIPVAKEA

>Hs_FMO_NP_001269621.1

MQENMAKRVAIVGAGVSGLASIKCCLEEGLEPTCFERSDDLGGLWRFTEHVEEGRASLYKSVVSNSCKEMSCYSDFPFPEDYPNYVPNSQFLEYLKMYANHFDLLKHIQFKTKVCSVTKCSDSAVSGQWEVVTMHEEKQESAIFDAVMVCTGFLTNPYLPLDSFPGINAFKGQYFHSRQYKHPDIFKDKRVLVIGMGNSGTDIAVEASHLAEKVFLSTTGGGWVISRIFDSGYPWDMVFMTRFQNMLRNSLPTPIVTWLMERKINNWLNHANYGLIPEDRTQLKEFVLNDELPGRIITGKVFIRPSIKEVKENSVIFNNTSKEEPIDIIVFATGYTFAFPFLDESVVKVEDGQASLYKYIFPAHLQKPTLAIIGLIKPLGSMIPTGETQARWAVRVLKGVNKLPPPSVMIEEINARKENKPSWFGLCYCKALQSDYITYIDELLTYINAKPNLFSMLLTDPHLALTVFFGPCSPYQFRLTGPGKWEGARNAIMTQWDRTFKVIKARVVQESPSPFESFLKVFSFLALLVAIFLIFL

>Mt_NMO_NP_216894.1

MNPTLAVLGAGAKAVAVAAKASVLRDMGVDVPDVIAVERIGVGANWQASGGWTDGAHRLGTSPEKDVGFPYRSALVPRRNAELDERMTRYSWQSYLIATASFAEWIDRGRPAPTHRRWSQYLAWVADHIGLKVIHGEVERLAVTGDRWALCTHETTVQADALMITGPGQAEKSLLPGNPRVLSIAQFWDRAAGHDRINAERVAVIGGGETAASMLNELFRHRVSTITVISPQVTLFTRGEGFFENSLFSDPTDWAALTFDERRDALARTDRGVFSATVQEALLADDRIHHLRGRVAHAVGRQGQIRLTLSTNRGSENFETVHGFDLVIDGSGADPLWFTSLFSQHTLDLLELGLGGPLTADRLQEAIGYDLAVTDVTPKLFLPTLSGLTQGPGFPNLSCLGLLSDRVLGAGIFTPTKHNDTRRSGEHQSFR

>Nf_NMO_WP_099421877.1

MNRRVRDSGRDDGYGRRTALAREWESGVETLLVVGAGPKALAVAAKSHVLRQLGLSAPRVIAVEAHAVGGNWLASGGWTDGRHRLGTSPEKDIGFPYHSTWARGHNREINEAMMAFSWTSFLVEHGTYAEWIDRGRPSPQHHVWAKYLQWVARKIDLELVLGKVRTIRQRPTDGGAGWSVEVAGADGATTELEADGLMITGPGQSTKALAKHPRVLSIAEFWDLAGKRKLPISSRAAVIGGGETAGSALDELVRHEMLTISVISPMATIYTRGESYFENSLFSDPTKWNALSIQERRDVIRRTDRGVFSVRVQESLLGDNRVHHLQGRVTRIVGQGDGVAVTLRNEMRADQVHNFDLVVDATGGQPLWFLDLFDSESADLLELAVGGPLTQQRIESSIGYDLAVTGLGAKLYLPNMAALAQGPGFPNLSCLGELSDRVLRAEPARVRAGARQLAAQ

>Pa_NMO_NP_251076.1

MTQATATAVVHDLIGVGFGPSNIALAIALQERAQAQGALEVLFLDKQGDYRWHGNTLVSQSELQISFLKDLVSLRNPTSPYSFVNYLHKHDRLVDFINLGTFYPCRMEFNDYLRWVASHFQEQSRYGEEVLRIEPMLSAGQVEALRVISRNADGEELVRTTRALVVSPGGTPRIPQVFRALKGDGRVFHHSQYLEHMAKQPCSSGKPMKIAIIGGGQSAAEAFIDLNDSYPSVQADMILRASALKPADDSPFVNEVFAPKFTDLIYSREHAERERLLREYHNTNYSVVDTDLIERIYGVFYRQKVSGIPRHAFRCMTTVERATATAQGIELALRDAGSGELSVETYDAVILATGYERQLHRQLLEPLAEYLGDHEIGRDYRLQTDERCKVAIYAQGFSQASHGLSDTLLSVLPVRAEEISGSLYQHLKPGTAARALHEHALAS

>Af_NMO_XP_755103.1

MESVERKSESSYLGMRNMQPEQRLSLDPPRLRSTPQDELHDLLCVGFGPASLAIAIALHDALDPRLNKSASNIHAQPKICFLERQKQFAWHSGMLVPGSKMQISFIKDLATLRDPRSSFTFLNYLHQKGRLIHFTNLSTFLPARLEFEDYMRWCAQQFSDVVAYGEEVVEVIPGKSDPSSSVVDFFTVRSRNVETGEISARRTRKVVIAIGGTAKMPSGLPQDPRIIHSSKYCTTLPALLKDKSKPYNIAVLGSGQSAAEIFHDLQKRYPNSRTTLIMRDSAMRPSDDSPFVNEIFNPERVDKFYSQSAAERQRSLLADKATNYSVVRLELIEEIYNDMYLQRVKNPDETQWQHRILPERKITRVEHHGPQSRMRIHLKSSKPESEGAANDVKETLEVDALMVATGYNRNAHERLLSKVQHLRPTGQDQWKPHRDYRVEMDPSKVSSEAGIWLQGCNERTHGLSDSLLSVLAVRGGEMVQSIFGEQLERAAVQGHQLRAML

>Ktz_NMO_WP_043726233.1

MTVAHAGESPTHDVVGVGFGPANLSLAVALEESPAALTSAFFERRASISWHQGMLLPAAKMQVSFLKDLATFRNPASRFSFVSFLHERGRLVRFANNHDFFPTRREFHDYLEWAESKLAHEVSYDSEVTAIRPGPGRPVDSVLVDVSTPEATRTVEARNIVISTGLVPRMPAGVQSDEFVWHSSRFLDHFRDRDPRSLRRVAVAGGGQSAAEIVRFLHDNRPDTVVHAIMPSYGYVVADNTPFANQIFDPAAVDDYFDGSKQAKDAFWRYHRNTNYSVVDDEVIRDLYRRGYDDEVAGAPRLNFVNLAHVVGAKRIADDTRVTVYSMAREESYDLDVDVLVCATGYDPMDPGDLLGELAEHCVQDAEGRWQVDRDYRMVTTPDLRCGIYLQGGTEHTHGLSSSLLSNLATRSGEIVSSIERRKS

>Ec_NMO_AAB71391.1

MKKSVDFIGVGTGPFNLSIAALSHQIEELDCLFFDEHPHFSWHPGMLVPDCHMQTVFLKDLVSAVAPTNPYSFVNYLVKHKKFYRFLTSRLRTVSREEFSDYLRWAAEDMNNLYFSHTVENIDFDKKRRLFLVQTSQGEYFARNICLGTGKQPYLPPCVKHMTQSCFHASEMNLRRPDLSGKRITVVGGGQSGADLFLNALRGEWGEAAEINWVSRRNNFNALDEAAFADEYFTPEYISGFSGLEEDIRHQLLDEQKMTSDGITADSLLTIYRELYHRFEVLRKPRNIRLLPSRSVTTLESSGPGWKLLMEHHLDQGRESLESDVVIFATGYRSALPQILPSLMPLITMHDKNTFKVRDDFTLEWSGPKENNIFVVNASMQTHGIAEPQLSLMAWRSARILNRVMGRDLFDLSMPPALIQWRSGT

>Gr_NMO_AOR50757.1

MTRVENVDVLAIGCGPFNLGLAALASTVDDLDVLVVDSREEFRWHPGLMFDEARLQVGFLSDLVTLVDPTHPMSFLNYMADTDRMYRFLVRENFYPTRIEYEAYLHWCIDRLDSLRWGTTVTEVSWDDNADAFAVTVVTGGTPSTVIARHVVVGVGTEPLVPESLSSDSPAVVHSSDYLYHQDKAHAADTVTVIGSGQSGAEIVIDLLEANLRGGPSVRWWTRTPWFAPLDFTKMSLEMTTPAYMDYFQSLPEEARDRIRPQHWQFHKGVSSDTLERVHELMYQRQLRDKLNPVQLRISTEVDGIDTLADGRLKVRGRHLDTGTQLAHTTDMVIACTGYQPRPMPFLAPIESQLHRDSRGRLVVGAAHQVETEPALANRLFVANAEEHAVGVSAPNLDIGAVRNARILNAVTGREVYRLPKDTAFTAFGVDDLDDVVG

>Ea_NMO_CBA23306.1

MNNTIYDFIGIGIGPFNLGLACLSEPVEGLNGVFLDQNPGFDWHTGMMLESAHLQTPFMADLVTLADPTSPYSLLNFMKQKGKLYSFYIREDFFLMRKEYNQYCQWAAERLGNLRWNTRVEYVSYDDNLQCYRVRSTDTVSGKQQEWLAHRLVLGTGPSAWSPACSQPYRERFVHSSEYLLNKEKLQKKRSITVLGSGQSAAEIYYDLLTDIDRFGYQLNWITRAPRFYPLEYTKLTLEMTSPEWIDYFHSLPAAKRDELNASQKNLYKGINSSLINAIYDLLYVKQLDGKLDVNLFTHSELTDMRWLAEGEFELKLHQQEQDRAYSRRTEGLVMATGYHYQPPAFVEGIQQRIQWDEKDRYDVQRNYSIDRHNQVFVQNAELHTHGFVTPDLGMACYRNSVLLREITGREVYPVERQIAFQTFPAQSEM

>Bp_NMO_CFO04355.1

MLRQGGIRDRHSKGESNMNREIYDFVAIGIGPFNLSLASLSAPLRGVRTLFLDKKSGFDWHPGMLIETSTLQNPFLADLVSLADPRSEYSYLNYCKLTNRIYSYYMRENHYLSRAEYTRYCQWVAARLPNLRFGCDVQGVLHDPESHSYLVTGQHTMSGQRFMFRCRKLVLGLGSQPYLPACCDRRAAPFIHSADYLRHKYELQGRASITIVGSGQSAAEVFHDLLRESGRHDYSLAWITRSPRFFQMENTKLTLELISPDYTEYFHDLPEARRQEILTQQNSLYKGINASLINQIYDLLDEKVHDGDNRYTLLTNSELRACRYDPLQERFQLDFQHLDCDRPFSHATDGLVLATGYSHEIPACINPIHDRIAWNADGSYRIGRNYAIDHEGSEIFVQNTGLLSHGVTNPDLGFCCYRNSQILRELTGTEHYRIETRTALQEFSPPADGVLKHRPARRAERRPTVAARPLMDIHRATL

>Str_NMO_AGJ55094.1

MTALADPRTAPYDFIGIGLGPFNLGLACLTEPIEELSGLFLESKPDFEWHSGMFLEGAHLQTPFMSDLVTMADPTSPYSFLNYLKARGRLYSFYIRENFYPLRTEYNDYCRWAAAELSSIRFNQTAQSVTYDEGDGLYTVRTADSAFRARHLVLGTGTPPYVPEACQGLGGDFLHNSRYLREKANLQAKESITLVGSGQSAAEIYHDLLSDIDAHGYRLNWVTRSPRFFPLEYTKLTLEMTSPEYVDYFHALPEDTRYRLESGQKSLFKGIDGELIDAIFDLLYQKNLHGPVATRLLTNSALRSASHDASTGTYTLGLRQEEQGRNYELHSEGLVLATGYRYTEPAFLEPVKDRIRYDSRGRFDVARNYSIDTTGRGIFLQNAGVHTHSITSPDLGMGAYRNAYIIGELLGREYYPVEKSVAFQEFAA

>Sb_NMO_ACK46161.1

MTTQQREMDREIFDLLGIGIGPFNLGLAALSEPIDGFNCLFLDAKPSFDWHPGMLLKSSRLQTPFMSDLVTMADPTSRYSYLNFAKKTGRLYPFYIRENFFLPRHEYNLYCQWVSQQLSNLKFGFKVTQVDYNAGESIYRVTGFDRRSGKTHTYQCRKLVLGTGTEPYLPKDCPIQDARVMHTASYMQQKTYLQSQSAITVIGSGQSAAEVFYDLLQDIDTYGYQLNWMTRSARFYPLEYTKLTLEMTSPDYVDYFHELAPEKRCALIASQKSLYKGINAELINDIYDLLYQKRLIADFQCQLMTNVALTRIQTDPDALKLHFQHQEQDAPLSQTTGAVVLGTGYHYRLPQFIKEIKQQIEFDDAGQLAVQRDYSIDIRGDIFVQNVGLHTHGVSSPDLGMGCYRNGIILKAVLGYAPYHIEEHIAFQTFDPAKLANHQPCGSNAINTLNLNPEHFPKTSPHTNIADASPHLYPTQTPTSSMSAGAGTKMSVLMAPNKEAQ

>SpCN32_NMO_ABP76132.1

MTTLQREIDREIFDLLGIGIGPFNLGLAALSEPIDGFNCLFLDAKTSFEWHPGMLLKSSRLQTPFMSDLVTMADPTSRYSYLNFAKQTGRLYPFYIRENFFLPRHEYNLYCQWVSQQLSNLKFGFKVTQVDYNAGEGIYRITGFDRRSGKTHTYQCRKLVLGTGTEPYLPKDCPIQDARIMHTASYMQQKTYLQSQSAITVIGSGQSAAEVFYDLLQDIDTYGYQLNWMTRSPRFYPLEYTKLTLEMTSPDYVDYFHELTPNQRCSLIASQKQLYKGINSELINDIYDLLYQKRLLADFQCRLMTNAALTHIEPQANMLKLHFHHNEKNHTFEQETGAVILGTGYHYRLPEFISGIKQQVEFDDAGQLSVQRDYSIDIRGDIFVQNVGLHTHGISSPDLGMGCYRNGTILKAVLGYAPYHIEEHIAFQTFDPAKLASHQPCGANAINTLNLKPKHFTKASDHTNTADAPQSLYPTQTATSSISAGTSSGAGTYMSVLMAPNKEAQ

>Sp200_NMO_ADV54887.1

MTTLQREIDREIFDLLGIGIGPFNLGLAALSEPIDGFNCLFLDAKTSFEWHPGMLLKSSRLQTPFMSDLVTMADPTSRYSYLNFAKQTGRLYPFYIRENFFLPRHEYNLYCQWVSQQLSNLKFGFKVTQVDYNAGEGIYRITGFDRRSGKTHTYQCRKLVLGTGTEPYLPKDCPIQDARIMHTASYMQQKTYLQSQSAITVIGSGQSAAEVFYDLLQDIDTYGYQLNWMTRSPRFYPLEYTKLTLEMTSPDYVDYFHELTPNQRCSLIASQKQLYKGINSELINDIYDLLYQKRLLADFQCRLMTNAALTHIEPQANMLKLHFHHNEKNHTFEQETGAVILGTGYHYRLPEFISGIKQQVEFDDAGQLSVQRDYSIDIRGDIFVQNVGLHTHGISSPDLGMGCYRNGTILKAVLGYAPYHIEEHIAFQTFDPAKLASHQPCGANAINTLNLKTKHFTKASNHTDTADAPHSLYPTQTATSSISAGTSSGAGTHMSVLMAPNKEAQ

>Sp95_NMO

MTTLQREIDREIFDLLGIGIGPFNLGLAALSEPIDGFNCLFLDAKTSFDWHPGMLLKSSRLQTPFMSDLVTMADPTSRYSYLNFAKQTGRLYPFYIRENFFLPRHEYNLYCQWVSQQLSNLKFGFKVTQVDYNAGEGIYRVTGFDRRSGKTHTYQCRKLVLGTGTEPYLPKDCPIQDARIMHTASYMQQKTYLQSQSAITVIGSGQSAADLFYDLLPDIDTYGYQLNWMTRSPRFYPLEYTKLTLEMTSPDYVDYFHELTPNQRCSLIASQKQLYKGINSELINDIYDLLYQKRLLADFQCRLMKNAALTHIEPQANMLKLHFHHNEKNHTFEQETGAVILGTGYHYRLPEFISGIKQQVEFDDAGQLSVQRDYSIDIRGDIFVQNVGLHTHGISSPDLGMGCYRNSTILKAVLGYAPYHIEEHIAFQTFDPAKLASHQPCGVNAINTLNLKTKHFTKASNHTDTADAPHSLYPTQTATSSISAGTSSGAGTHMSVLMAPNKEAQ

>Al_NMO_WP_039794392.1

MARAVFGERVPVYDVVGVGFGPSNLALAIAVTEHNAAPGAETVTAHFLERQACFGWHRGMLIDNATMQVSFLKDLATMRNPTSSFSFLSYLHSKDRLVDFINHKNLFPLRIEFHDYFEWAAEKVDDLVSYGTEVLSVTPVFDGDEIEFFDVHARTDGELVNLRARNLVMGTGLRPNLPEGVTPGTRVWHNSELLHRVEGMAAEEPRRFVVVGAGQSAAEVSALLHDRFPQAEVCAVFARYGYSPADDSAFANRIFDPEAVGRFYEAPEAVKDRLMRYHGATNYSAVDIDLIDELYRRVYR

EKVQGVERLRLINVSRPTEVVDTGSEVRVTVEALESGERTRIDADFVVYATGYSPADPTSLLGELASACARDDEG

RLRVERDYRIVTEPPLDGGIYLQGGTEHTHGITSSLLSNTAVRVGEILQSIVDRRVADASRPEYAVSGTGPA

| **Table S1. Percentage of amino acids present in the class B monooxygenases selected for the study** | | | | | | | | | | | | | | | | | | |
| --- | --- | --- | --- | --- | --- | --- | --- | --- | --- | --- | --- | --- | --- | --- | --- | --- | --- | --- |
| **Name of the amino acids** | **Name of the class B monooxygenase** | | | | | | | | | | | | | | | | | |
|  | **Sp95**  **_NMO** | **Sp200**  **_NMO** | **SpCN32**  **_NMO** | **Sb_NMO** | **Ea_NMO** | **Bp_NMO** | **Str_NMO** | **Gr_NMO** | **Ec_NMO** | **Mt_NMO** | **Nf__NMO** | **Pa_NMO** | **Af_NMO** | **Ktz_NMO** | **Al_NMO** | **Xf_BVMO** | **Ma_FMO** | **Hs_FMO** |
| **Ala** | 6.30% | 6.50% | 6.50% | 7.00% | 5.60% | 6.70% | 8.20% | 8.40% | 5.60% | 10.70% | 10.70% | 9.90% | 6.80% | 9.70% | 8.70% | 0.30% | 7.00% | 5.60% |
| **Arg** | 4.20% | 4.20% | 4.20% | 4.20% | 6.30% | 9.40% | 7.00% | 7.50% | 6.80% | 8.40% | 9.00% | 7.40% | 8.20% | 9.00% | 3.10% | 5.50% | 4.60% | 3.70% |
| **Asn** | 4.40% | 4.40% | 4.40% | 4.00% | 4.90% | 4.20% | 3.30% | 2.70% | 4.50% | 2.60% | 2.40% | 2.50% | 3.40% | 3.10% | 6.70% | 3.80% | 3.70% | 4.50% |
| **Asp** | 5.70% | 5.30% | 5.30% | 6.00% | 5.60% | 6.10% | 5.60% | 8.40% | 5.60% | 6.50% | 5.50% | 5.20% | 5.40% | 8.50% | 0.70% | 6.20% | 7.20% | 4.10% |
| **Cys** | 1.60% | 1.60% | 1.60% | 1.60% | 1.20% | 2.30% | 0.70% | 0.70% | 1.20% | 0.50% | 0.20% | 0.90% | 1.00% | 0.70% | 1.60% | 0.50% | 1.50% | 2.10% |
| **Gln** | 6.10% | 6.30% | 6.50% | 7.00% | 6.30% | 4.20% | 2.80% | 3.00% | 3.30% | 3.20% | 3.50% | 5.20% | 5.40% | 2.10% | 8.10% | 3.10% | 2.90% | 3.00% |
| **Glu** | 4.00% | 4.40% | 4.40% | 4.00% | 6.70% | 5.40% | 7.00% | 5.50% | 6.40% | 4.90% | 5.90% | 7.00% | 6.80% | 5.70% | 7.60% | 5.70% | 7.90% | 6.50% |
| **Gly** | 6.50% | 6.70% | 6.70% | 6.20% | 5.80% | 6.30% | 8.20% | 6.40% | 5.90% | 9.70% | 10.70% | 6.80% | 5.00% | 6.40% | 2.90% | 9.20% | 5.90% | 6.00% |
| **His** | 4.00% | 4.00% | 3.60% | 3.20% | 2.60% | 4.40% | 2.80% | 3.40% | 3.50% | 3.20% | 2.90% | 3.80% | 3.40% | 3.80% | 3.40% | 0.90% | 3.10% | 1.90% |
| **Ile** | 5.70% | 5.90% | 5.90% | 5.60% | 4.20% | 5.20% | 4.00% | 3.20% | 4.50% | 3.90% | 3.90% | 5.00% | 4.80% | 2.80% | 8.30% | 4.90% | 4.60% | 6.50% |
| **Leu** | 10.50% | 10.30% | 10.30% | 10.20% | 10.90% | 11.30% | 11.70% | 9.80% | 11.10% | 10.70% | 10.30% | 10.60% | 9.60% | 7.30% | 1.30% | 7.00% | 6.60% | 9.00% |
| **Lys** | 4.60% | 4.40% | 4.40% | 4.60% | 4.00% | 2.10% | 2.30% | 1.40% | 4.00% | 1.60% | 2.60% | 2.70% | 5.00% | 1.70% | 1.60% | 5.70% | 5.90% | 6.50% |
| **Met** | 2.60% | 2.60% | 2.60% | 2.80% | 2.60% | 1.70% | 1.40% | 2.50% | 3.30% | 1.20% | 1.80% | 1.60% | 3.00% | 1.90% | 4.70% | 2.60% | 3.10% | 2.80% |
| **Phe** | 5.00% | 5.00% | 5.00% | 4.80% | 4.70% | 4.00% | 5.10% | 3.70% | 6.10% | 4.20% | 2.40% | 3.80% | 3.40% | 5.00% | 10.10% | 5.30% | 5.50% | 6.90% |
| **Pro** | 4.80% | 4.60% | 4.80% | 5.60% | 4.20% | 4.80% | 4.90% | 5.30% | 4.90% | 4.40% | 3.90% | 4.10% | 5.60% | 5.00% | 4.70% | 4.20% | 4.80% | 6.00% |
| **Ser** | 6.90% | 6.70% | 6.70% | 6.20% | 6.00% | 7.10% | 7.00% | 5.50% | 8.00% | 6.00% | 6.60% | 7.20% | 9.60% | 8.00% | 6.00% | 4.20% | 5.50% | 7.10% |
| **Thr** | 7.70% | 7.90% | 7.70% | 7.00% | 5.30% | 5.20% | 7.00% | 7.50% | 5.20% | 7.70% | 5.30% | 4.70% | 4.00% | 5.00% | 5.60% | 8.10% | 6.10% | 5.20% |
| **Trp** | 0.60% | 0.60% | 0.60% | 0.60% | 2.10% | 0.80% | 0.70% | 1.80% | 1.90% | 2.30% | 2.60% | 0.50% | 1.00% | 1.20% | 0.70% | 2.00% | 3.10% | 1.90% |
| **Tyr** | 5.70% | 5.70% | 5.90% | 5.80% | 6.30% | 5.40% | 6.80% | 2.70% | 2.80% | 1.20% | 1.80% | 4.30% | 2.60% | 3.30% | 3.60% | 3.70% | 5.90% | 3.50% |
| **Val** | 3.20% | 3.00% | 3.00% | 3.80% | 4.90% | 3.30% | 3.50% | 10.50% | 5.40% | 7.20% | 7.90% | 6.80% | 6.20% | 10.10% | 10.70% | 7.10% | 5.00% | 7.30% |


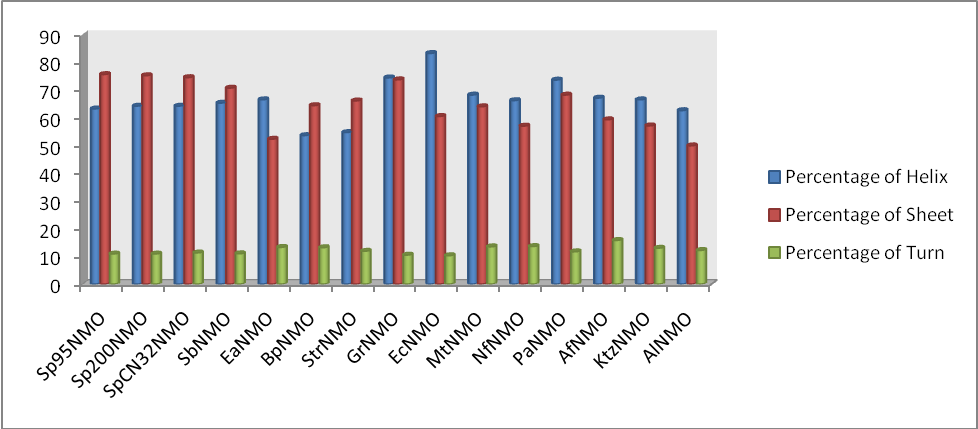
**Fig S2. Graphical representation of percentage of helices, sheets and turns of N-hydroxylating monooxygenases from the selected microorganisms**

**
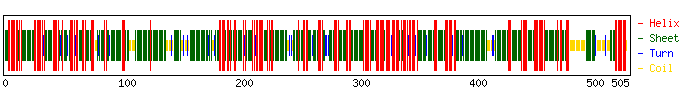
**

**Fig S3. Pictorial representation of secondary structure predicted using Chou Fasman algorithm.**

**
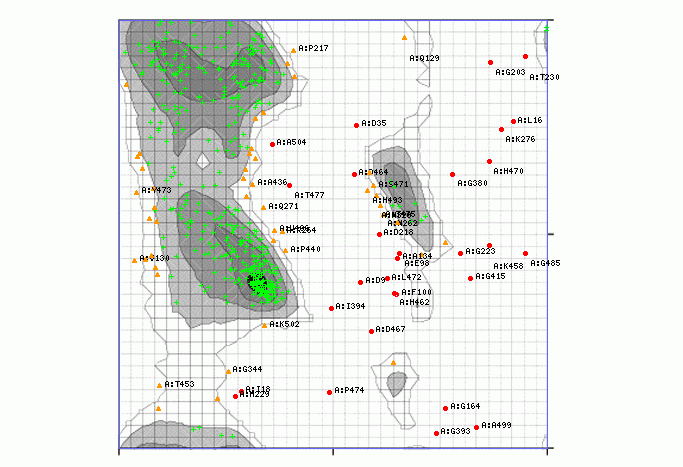
**

**Fig S4. Validation of 3D model using Ramachandran plot by ZLab server.**

| Black   | Dark Grey | Grey | Light Grey | represent Highly Preferred Conformations. Delta >= -2 |  | | --- | --- | --- | --- | --- | |
| --- | --- | --- | --- | --- | --- |
| White with **Black Grid** represents preferred conformations. -2 > Delta >= -4 |
| White with Grey Grid represents questionable conformations. Delta < -4 |
| Highly Preferred observartions shown as GREEN Crosses: 427 (84.891%) |
|  |
| Preferred observations shown as BROWN Triangles: 47 (9.344%) |
|  |
| Questionable observations shown as RED Circles: 29 (5.765%) |
| 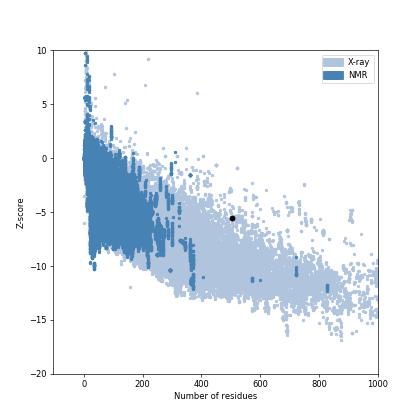*Z*-Score: **-5.53** |

**Fig S5: Overall model quality assessment by ProSA-web**


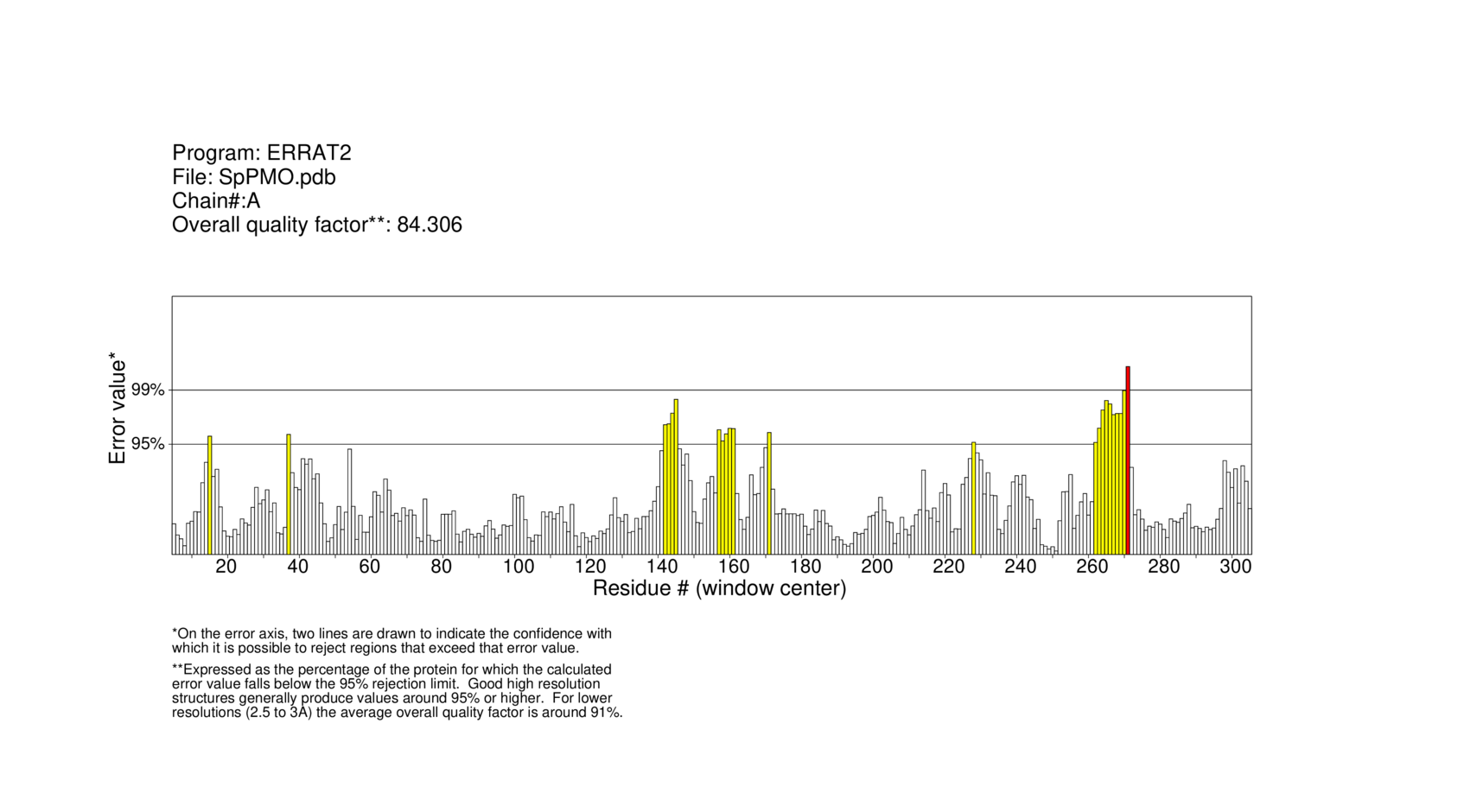

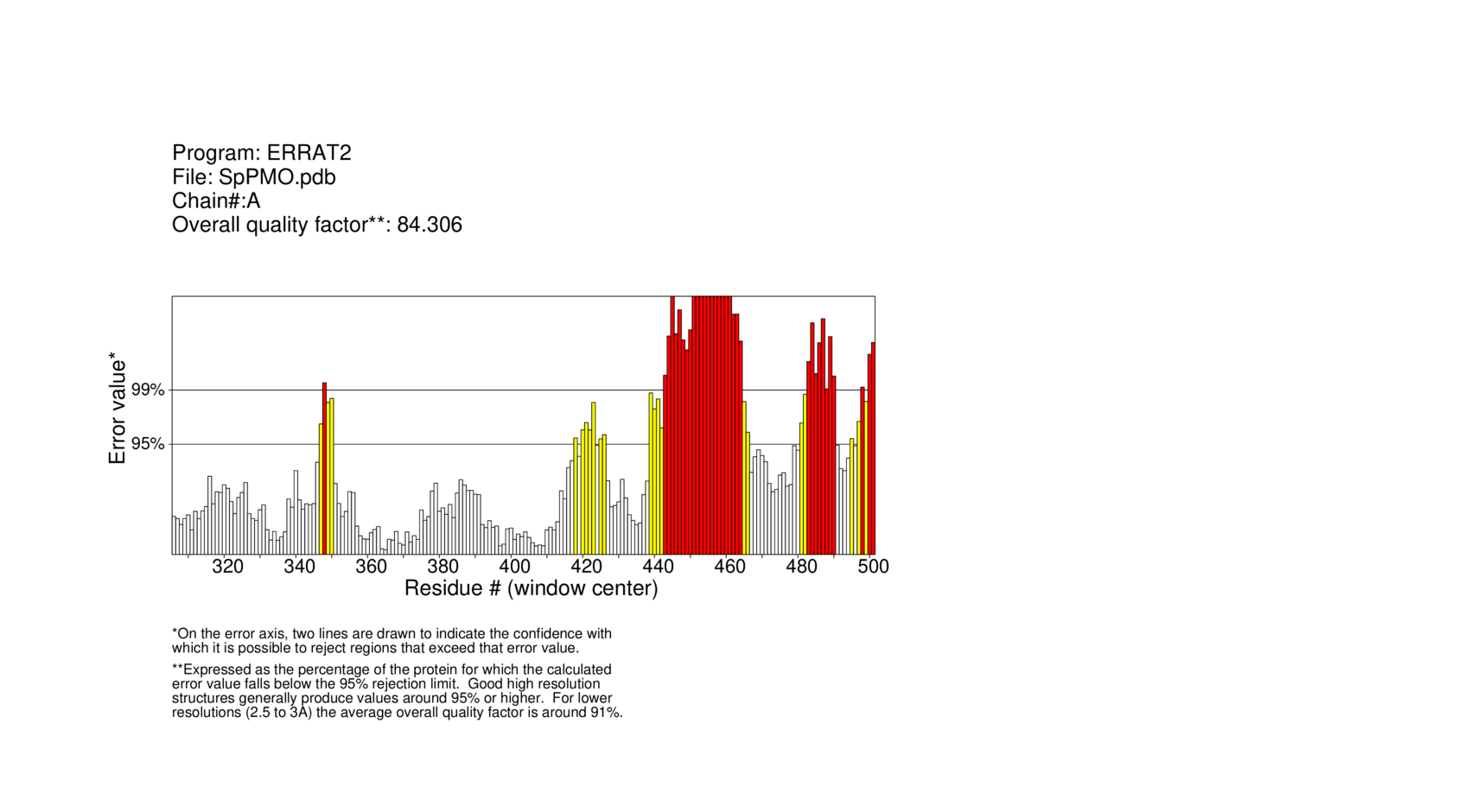


**Fig S6. Overall quality factor of *Sp*PMO95 protein assessed using ERRAT software in SAVES server and was found to be 84.306.**


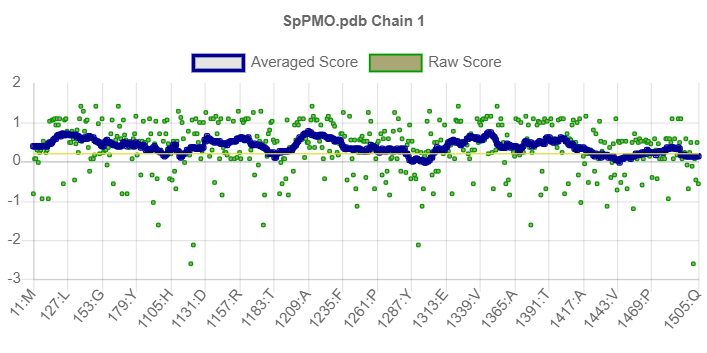


**Fig S7. The overall quality of *Sp*PMO95 assessed by Verify-3D in SAVES server showing 83.96% of the residues have averaged 3D-1D score >= 0.2.**

**
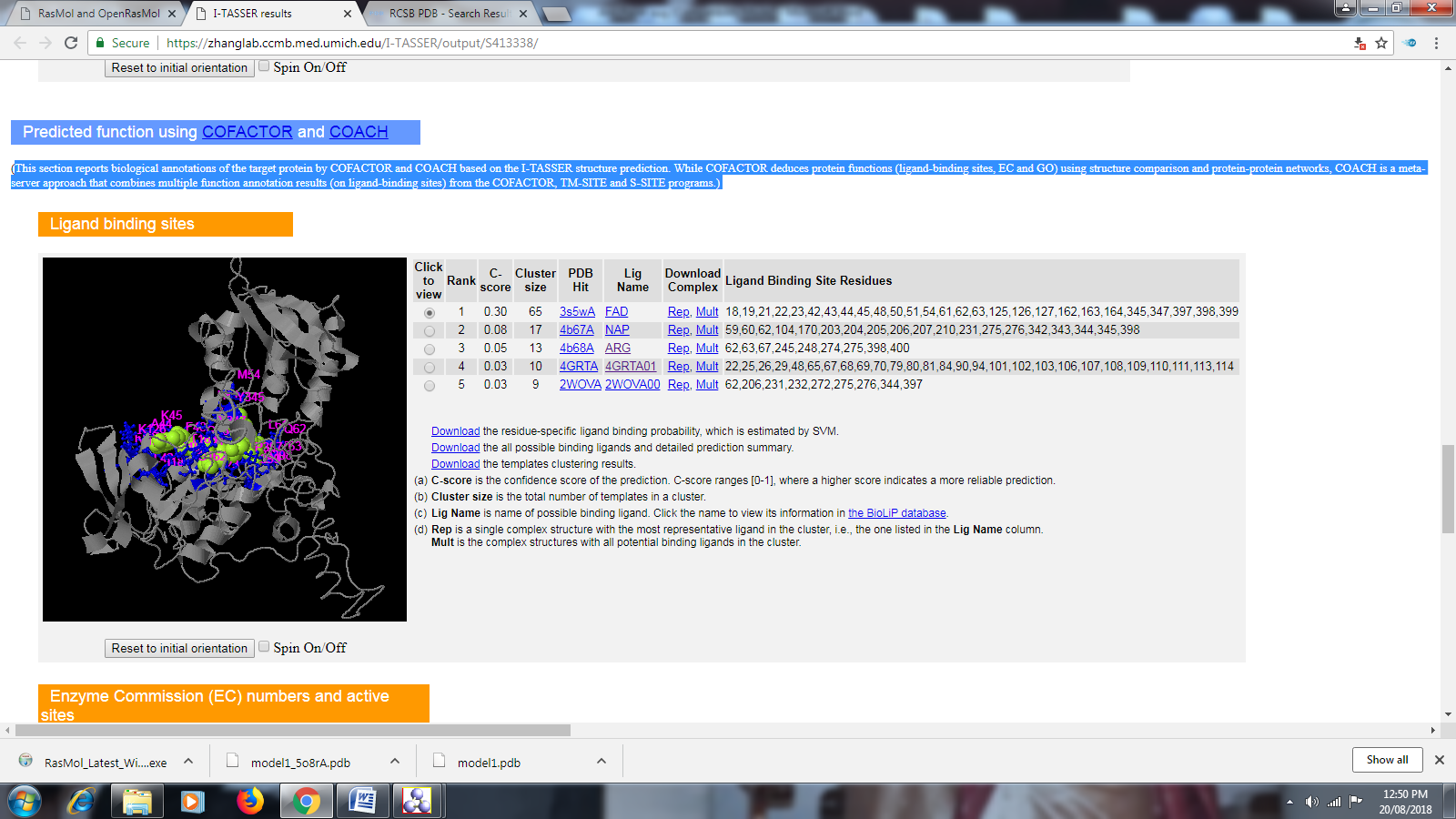
**

FAD binding sites - I18, G19, G21, P22, L23, D42, A44, K45, F48, W50, H51, M54, L61, Q63, T125, G163 Y345, Y347, P397, D398 and L399

**
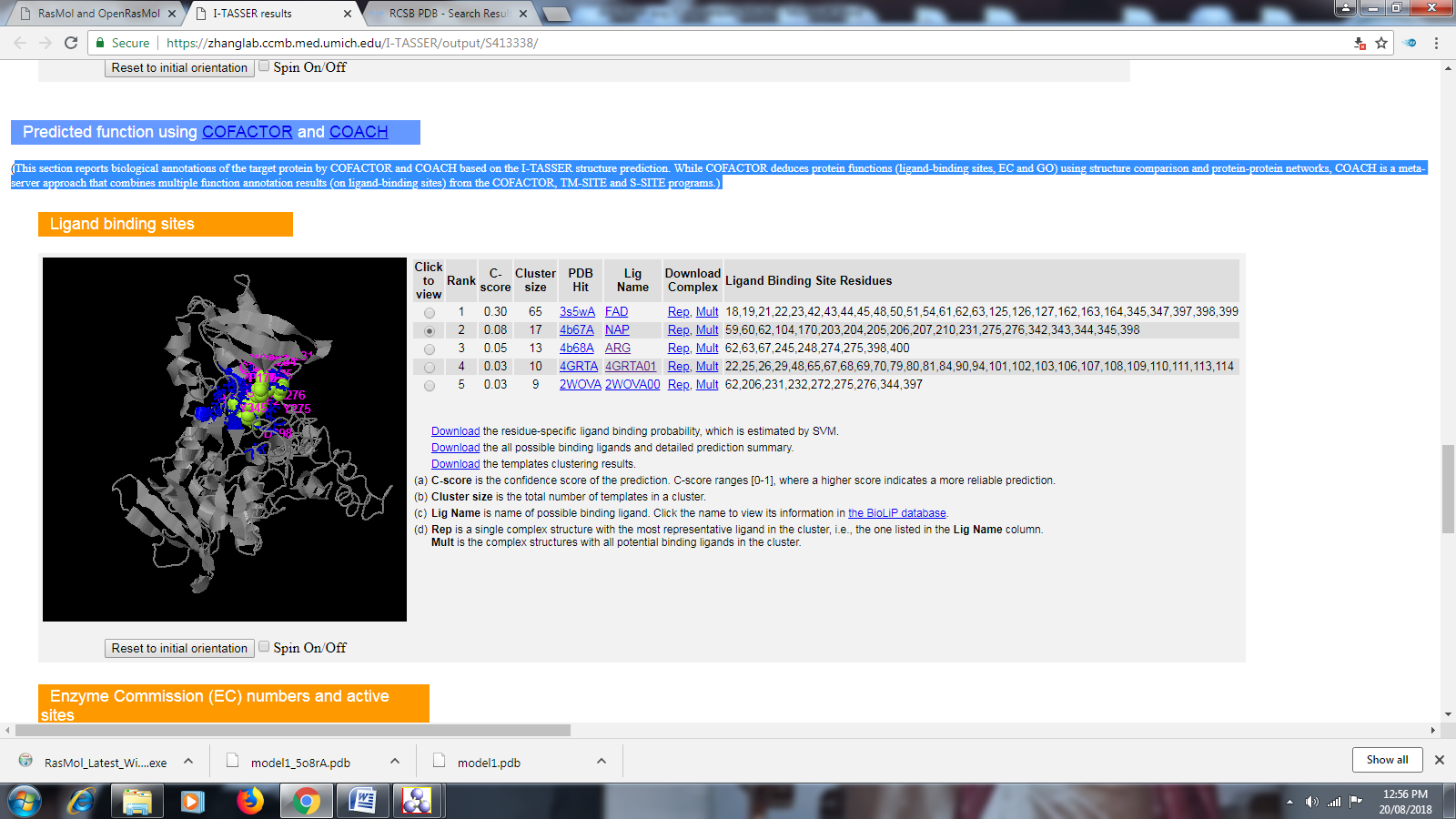
Fig S8. Predicted FAD binding site of *Sp*PMO, coloured in blue. FAD binding sites are given in the box.**

NAD binding sites -

S59, R60, Q62, R104, P170, G203, S204, 210D, R231, Y275, G342, T343, Y345

**Fig S9. Predicted NAD binding site of *Sp*PMO, colored in blue.NAD binding sites are given in the box.**


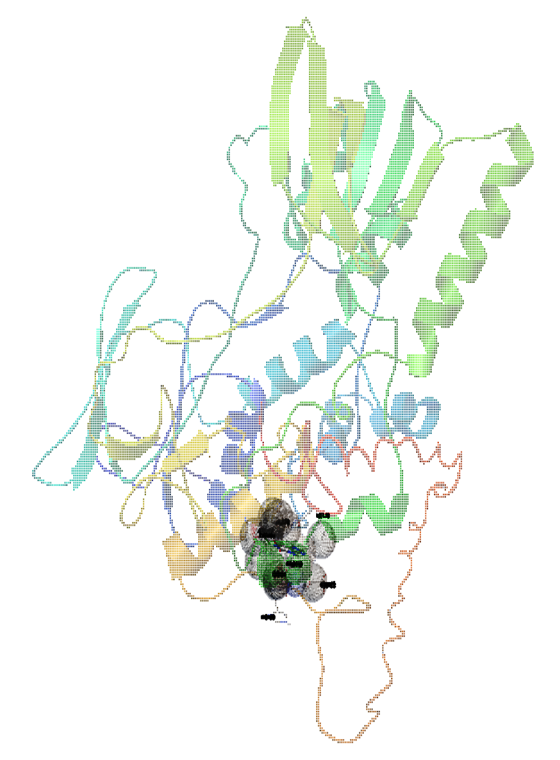


Vanilin

Caffeine

Theobromine

Theophylline

Allicin

Niazirin

Niazirinin

Capsaicin

Piperine

Piperine


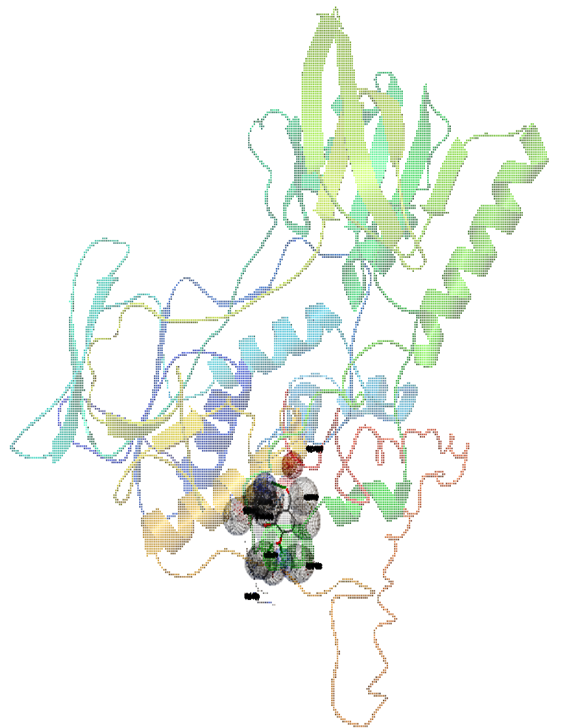

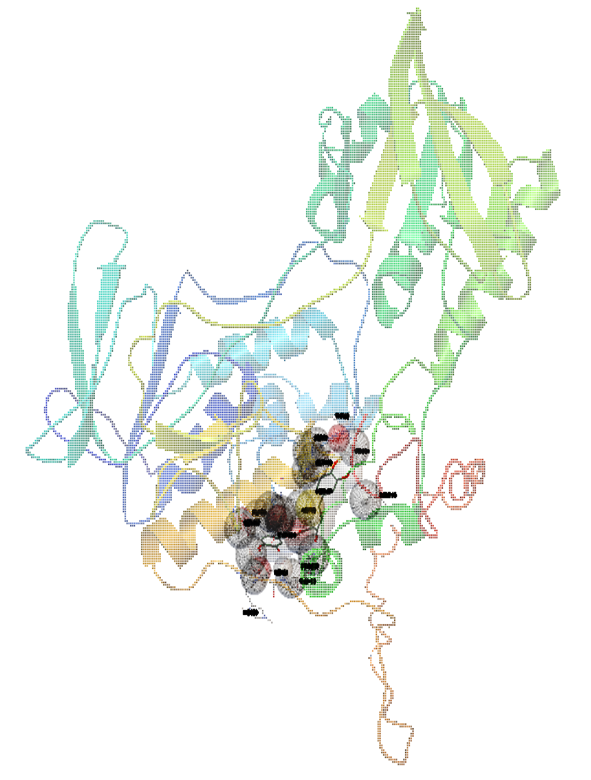


Curcumin


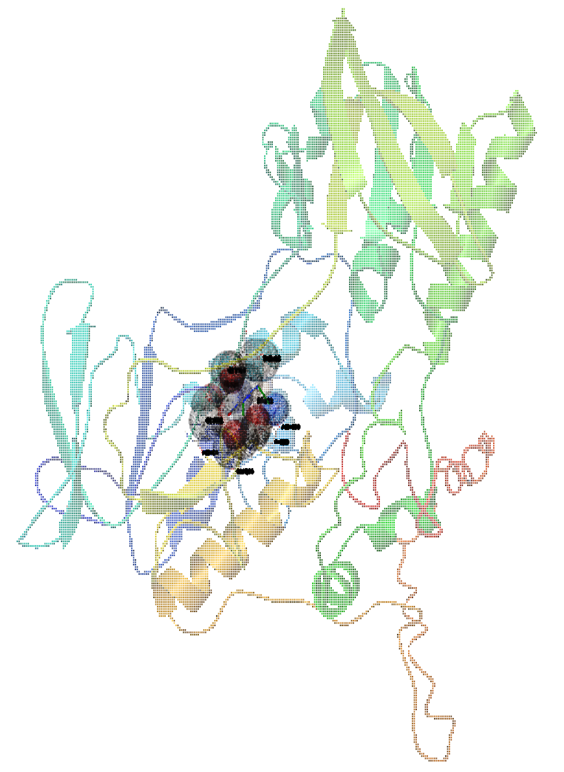

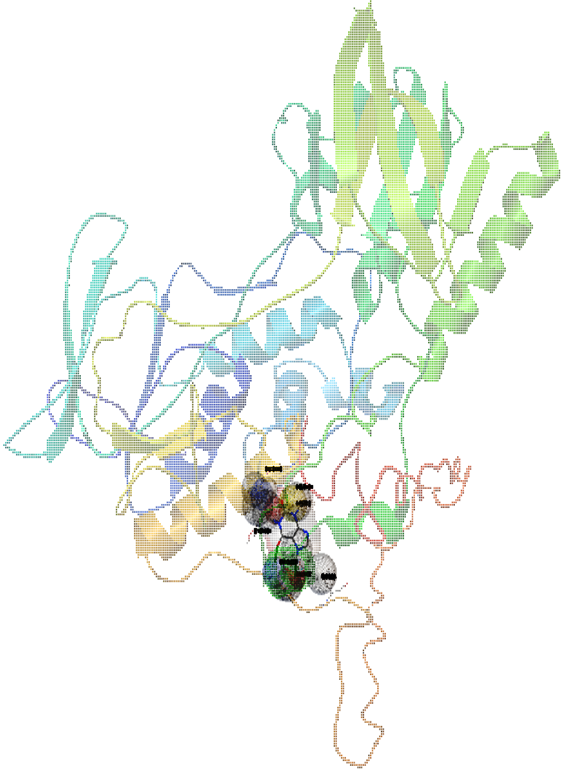

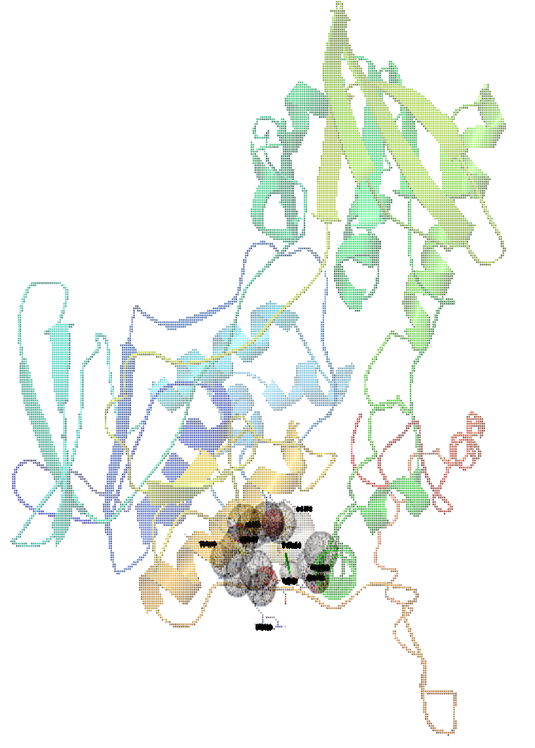

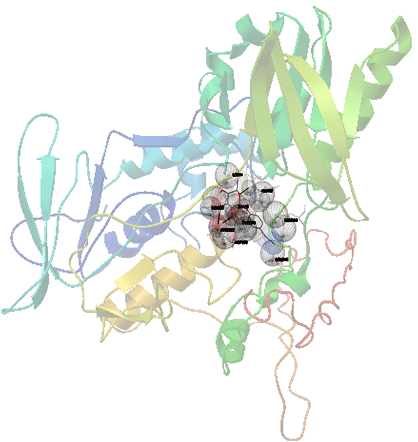

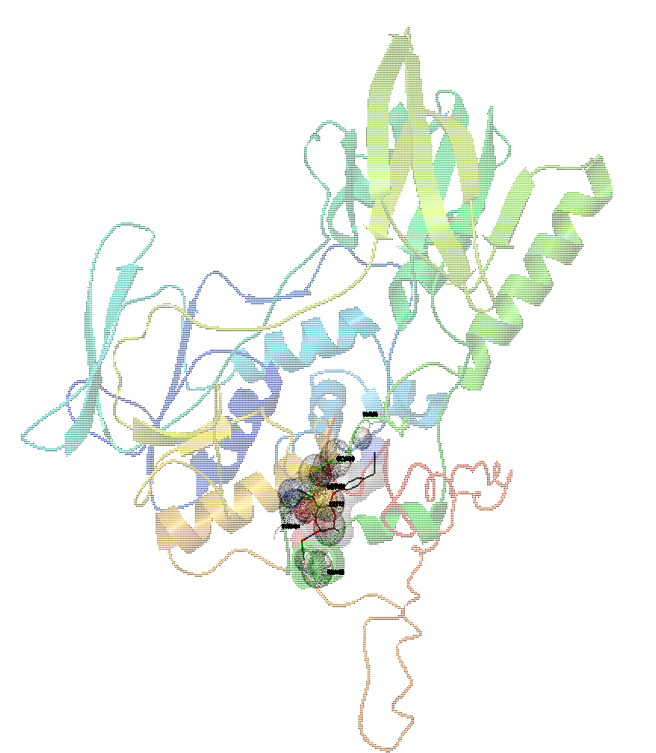

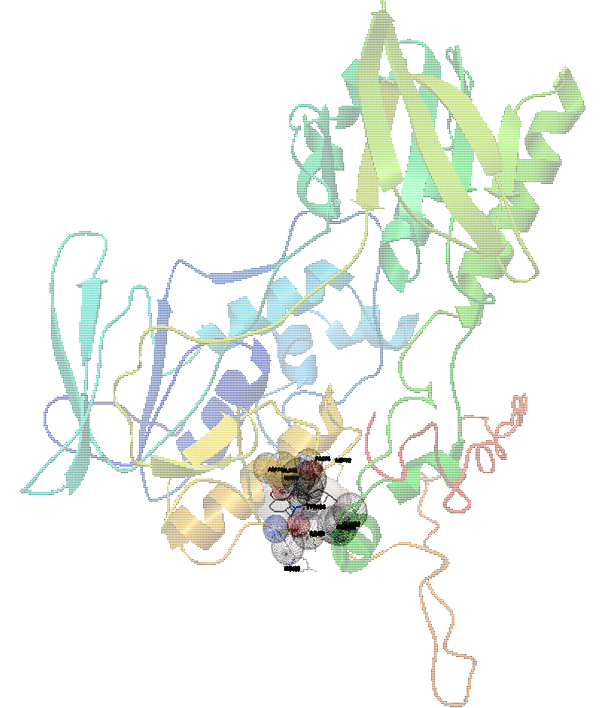

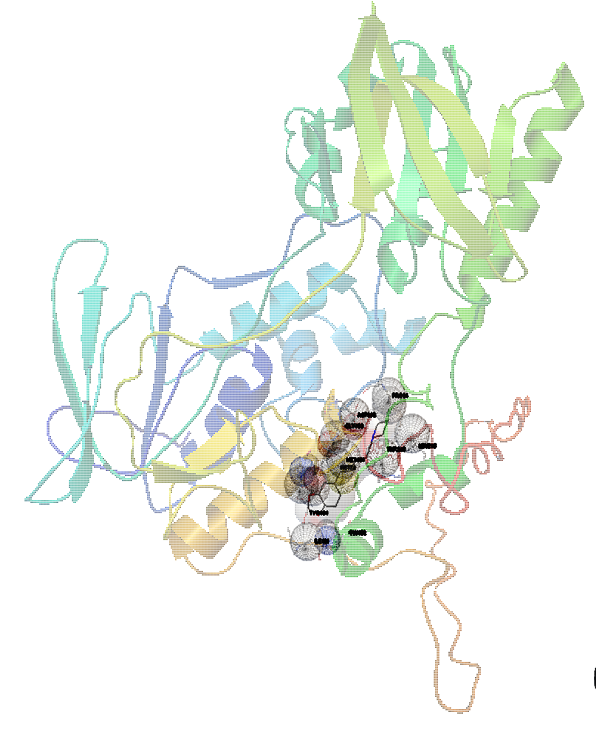

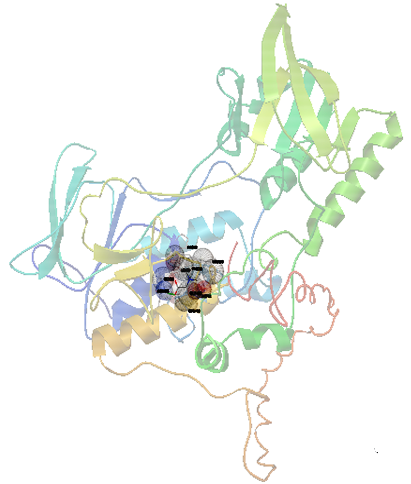


Betaine

Piperine


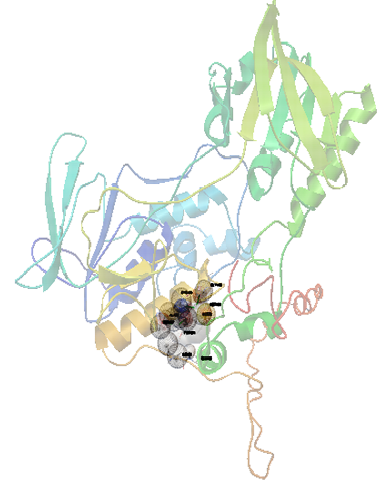


Dopamine

Piperine


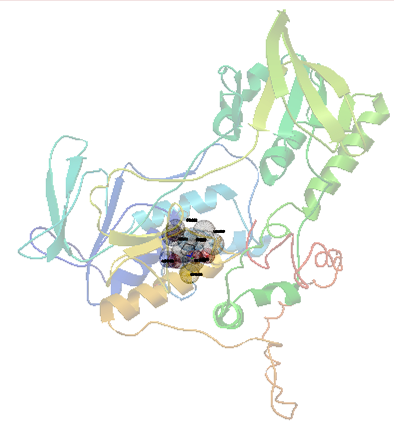


Piperidine


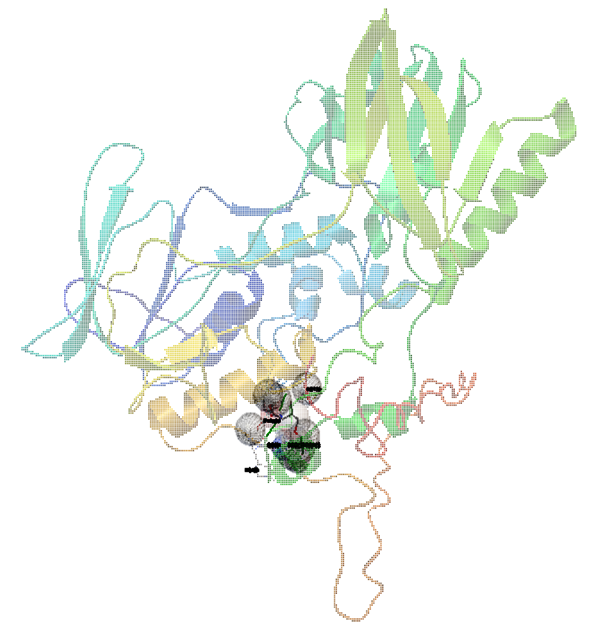


4-hydroxyleucine


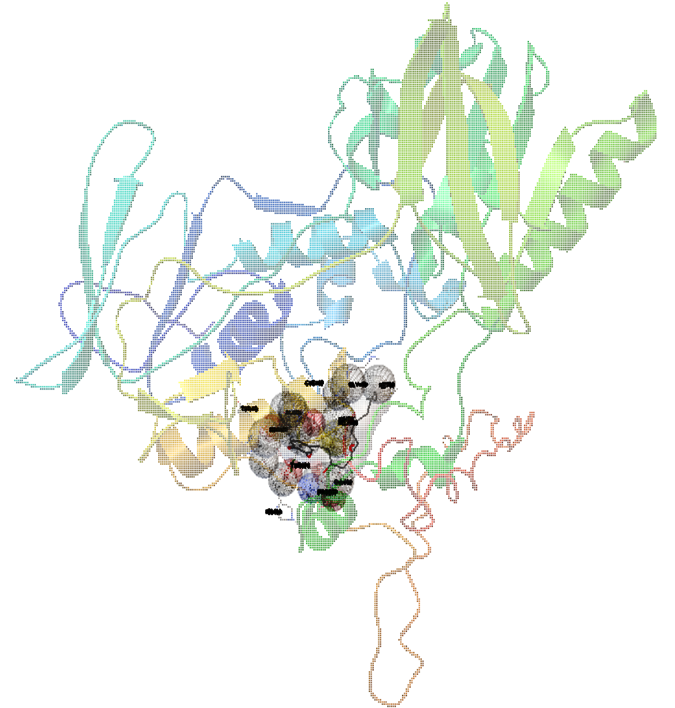


10-gingerol


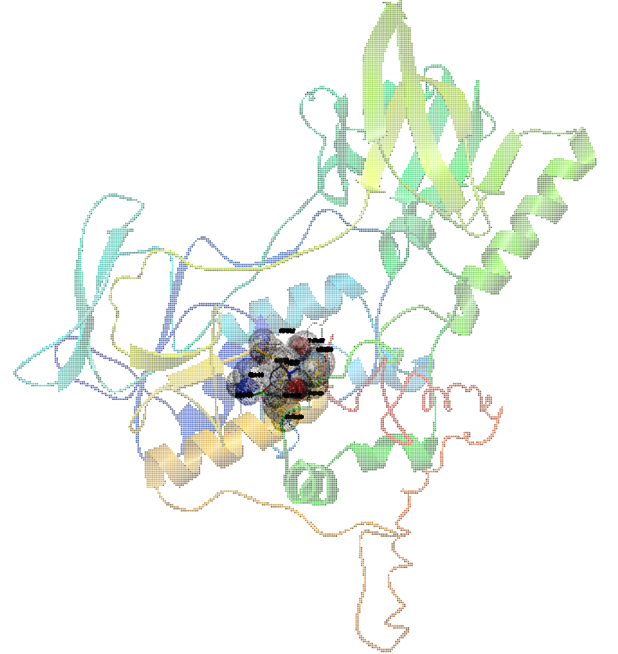


Tropine


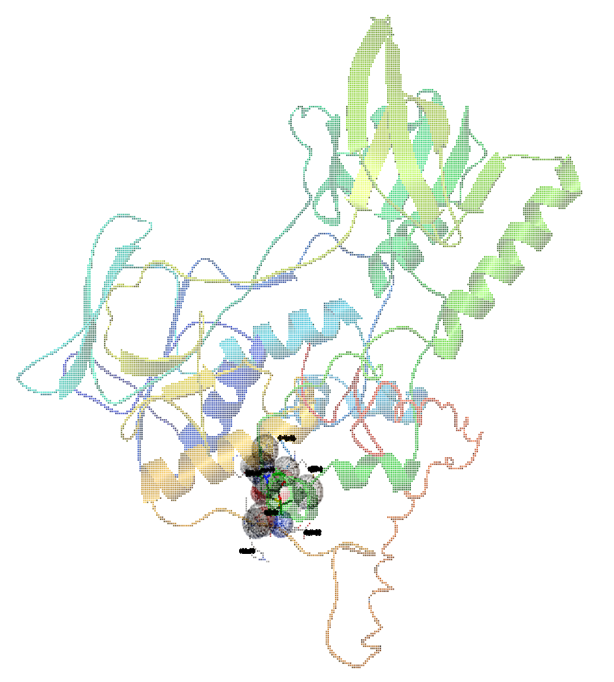


S-allyl-cysteine


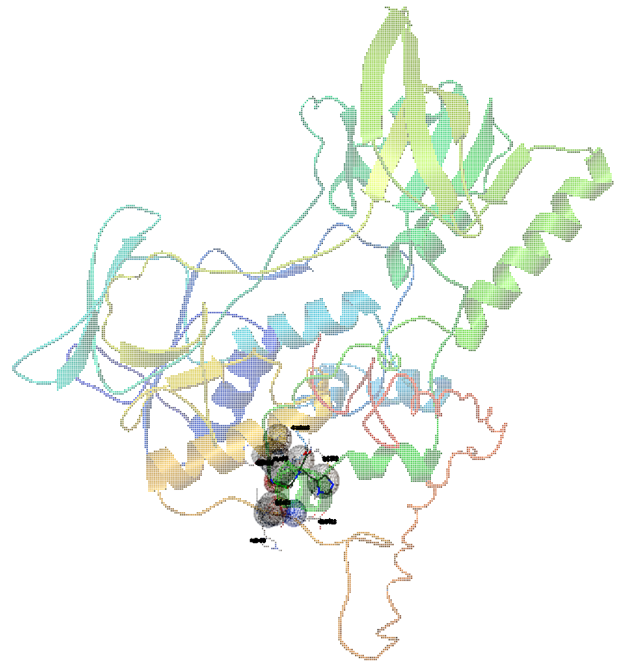


Carnosine


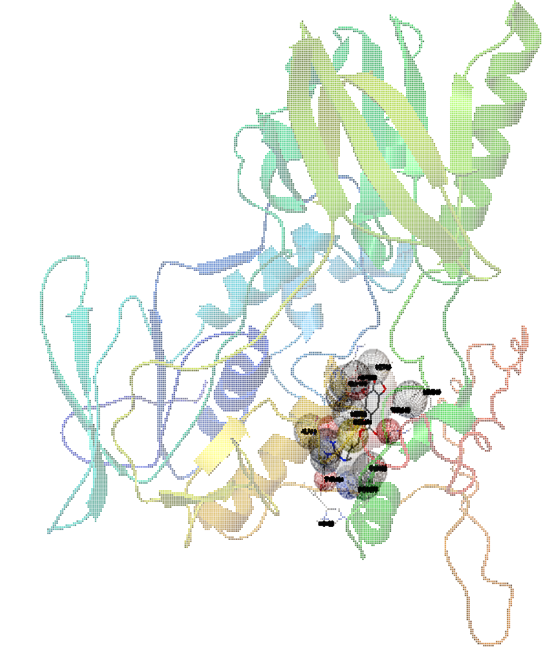


Leonurine


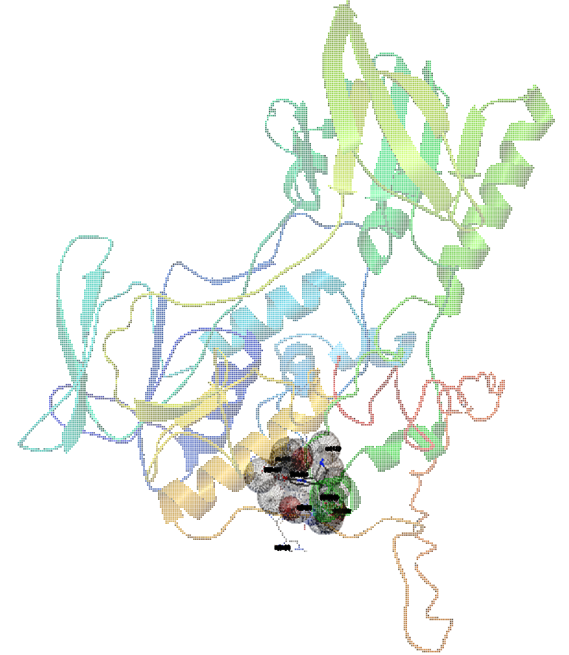


Cystisine


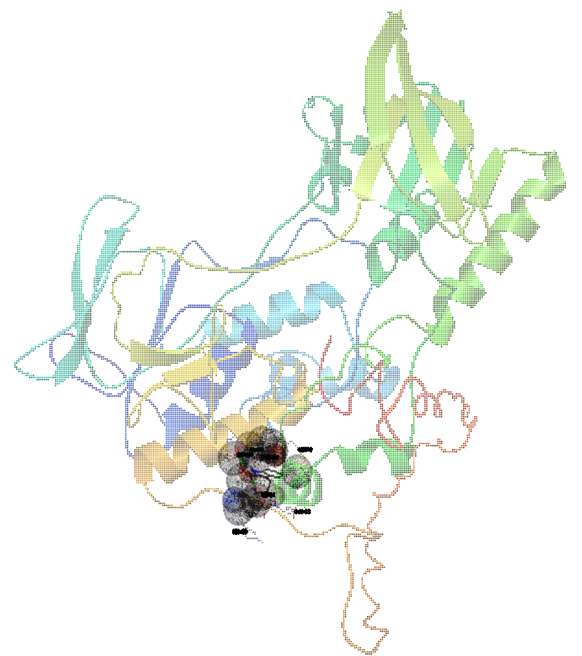


Tryptamine


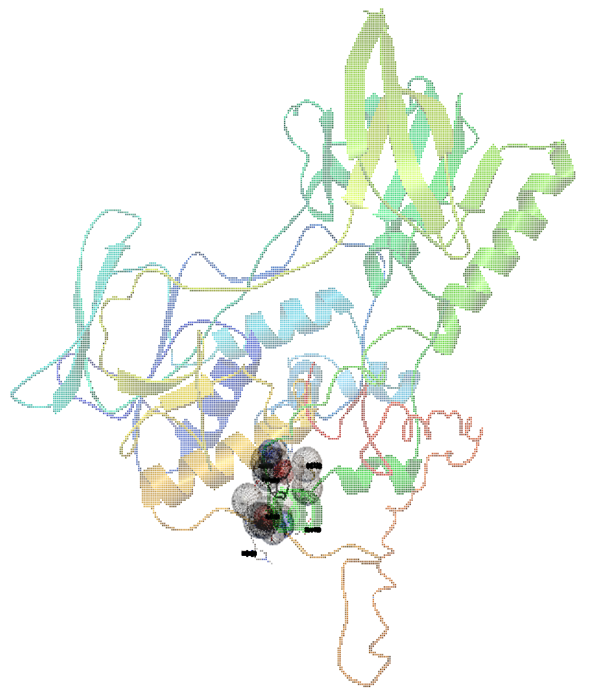


Beta-pinene


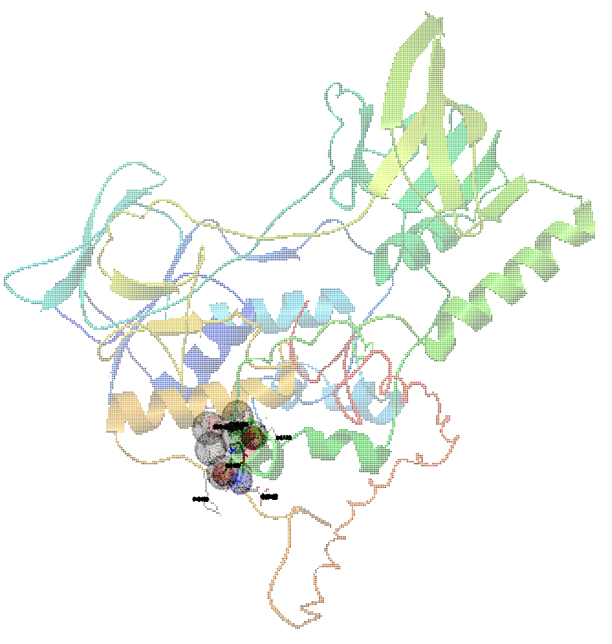


Beta-alanine


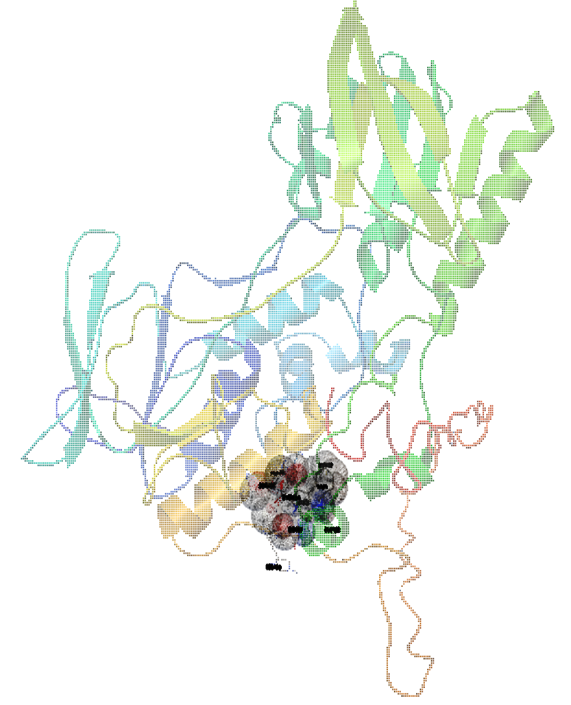


L-abrine


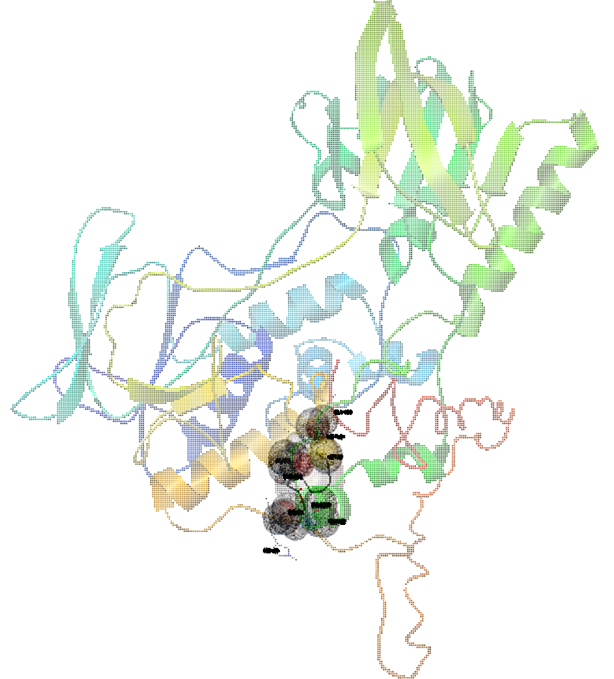

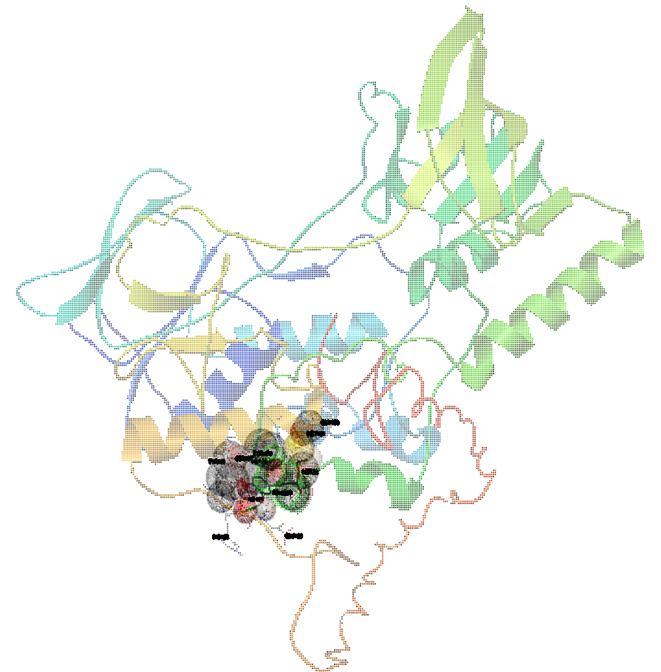

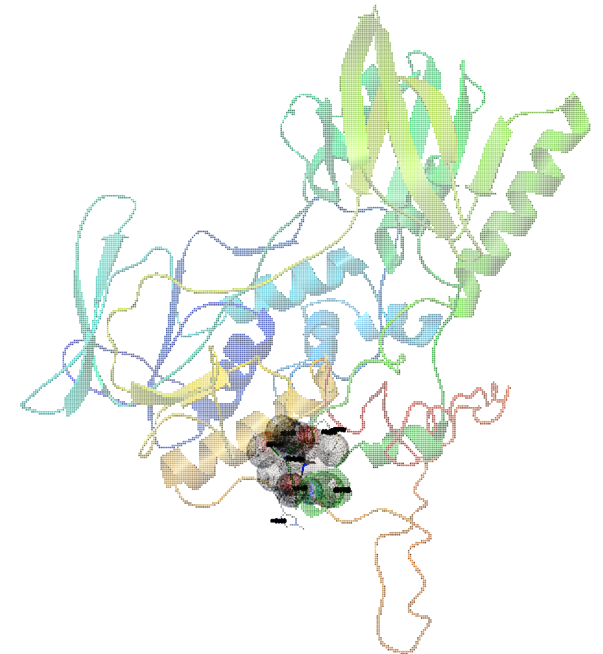

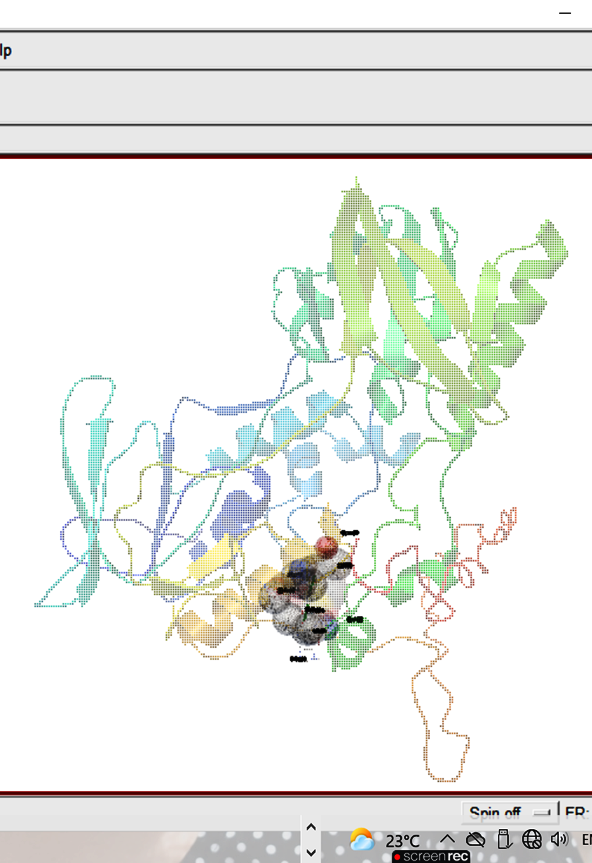


Undecanoic acid

Alliin

Ethyl caffeate

Gramine

**Fig S10. Pictures showing the interaction of *Sp*PMO and the respective inhibitors**.
